# Recruitment of trimeric eIF2 by phosphatase non-catalytic subunit PPP1R15B

**DOI:** 10.1016/j.molcel.2023.12.011

**Published:** 2023-12-29

**Authors:** Agnieszka Fatalska, George Hodgson, Stefan M.V. Freund, Sarah L. Maslen, Tomos Morgan, Sigurdur R. Thorkelsson, Marjon van Slegtenhorst, Sonja Lorenz, Antonina Andreeva, Laura Donker Kaat, Anne Bertolotti

**Affiliations:** 1 MRC Laboratory of Molecular Biology, Francis Crick Avenue, Cambridge CB2 0QH, United Kingdom; 2 Department of Clinical Genetics, Erasmus MC, University Medical Center Rotterdam, Rotterdam, the Netherlands; 3 Max Planck Institute for Multidisciplinary Sciences, Am Fassberg 11, 37077 Göttingen, Germany

## Abstract

Regulated protein phosphorylation controls most cellular processes. The protein phosphatase PP1 is the catalytic subunit of many holoenzymes that dephosphorylate serine/threonine residues. How these enzymes recruit their substrates is largely unknown. Here, we integrated diverse approaches to elucidate how the PP1 non-catalytic subunit PPP1R15B (R15B) captures its full trimeric eIF2 substrate. We found that the substrate-recruitment module of R15B is largely disordered with three short helical elements, H1, H2, and H3. H1 and H2 form a clamp that grasps the substrate in a region remote from the phosphorylated residue. A homozygous N423D variant, adjacent to H1, reducing substrate binding and dephosphorylation was discovered in a rare syndrome with microcephaly, developmental delay, and intellectual disability. These findings explain how R15B captures its 125 kDa substrate by binding the far end of the complex relative to the phosphosite to present it for dephosphorylation by PP1, a paradigm of broad relevance.

## Graphical abstract

**Graphical abstract:**
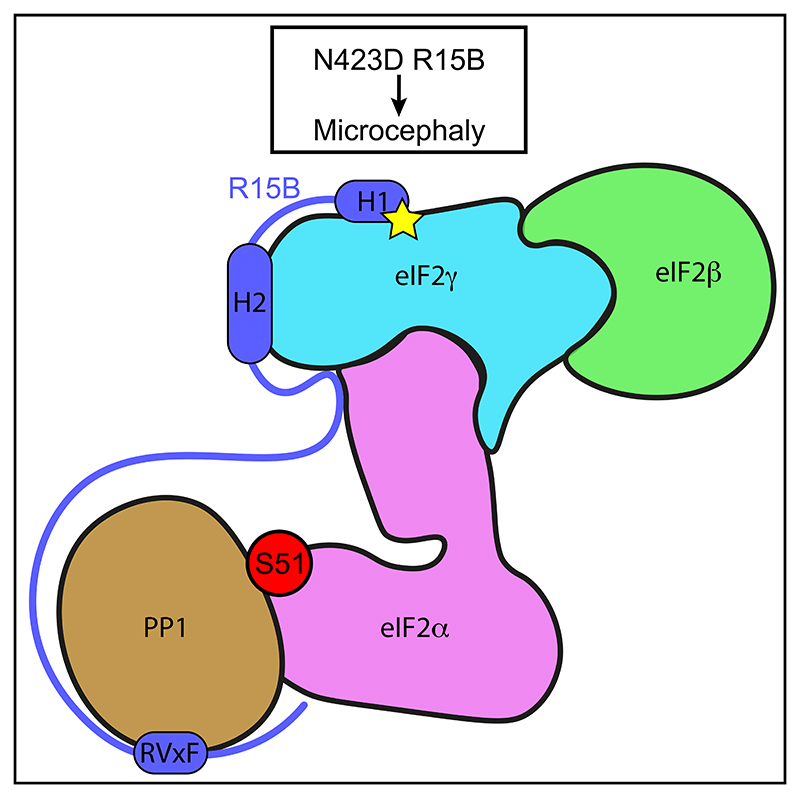

## Introduction

Reversible phosphorylation of proteins is one of the most prevalent post-translational modifications that allows cells and organisms to rapidly adapt to changes, an essential requirement for survival and fitness.^1^ In mammals, this occurs most commonly on serine and threonine residues and is catalyzed by opposing protein kinases and phosphatases.^2^ More than 500 kinases sense changes in the environment and phosphorylate an estimated one million sites in mammalian proteins,^2^ and dephosphorylation is achieved by several hundred phosphatases.^3^

PP1 is a major serine/threonine phosphatase^1,4,5^ with a catalytic mechanism that was revealed when its three-dimensional structure was determined.^6,7^ The active site, containing two metal ions, is located at the trifurcation point of a shallow Y-shaped groove.^6,7^ These metal ions activate a water molecule for nucleophilic attack on the phosphate.^6,7^ Unlike serine/threonine kinases, which possess a peptide-binding cleft important for substrate selectivity, PP1 has only a shallow Y-shape groove near its catalytic site.^6,7^ This groove may enable broad selectivity of PP1 for a large number of substrates.

In cells, PP1 exists in complex with one of hundreds of non-catalytic subunits, thus forming a repertoire of holoenzymes.^5,8–10^ How PP1 is recruited to its non-catalytic subunits is well understood. The first structure of a 13 residue-fragment of a non-catalytic subunit bound to PP1 identified the RVxF binding motif in glycogen-binding protein GM.^11^ This motif turned out to be common to most non-catalytic subunits.^5,8–10^ Structural analyses of minimal PP1-binding fragments of several non-catalytic subunits identified other short linear motifs that provide additional binding sites such as the MyPhoNE motif in MYPT1,^12^ the SILK motif in inhibitor-2 and ASPP,^13,14^ and the φφ motif in NIPP1, spinophilin, and PNUTS.^15–17^ These conserved short linear motifs provide a combinatorial and generic repertoire of docking sites to PP1.^18^ The interactions between PP1 and these docking sites are identical for many non-catalytic subunits. Thus, selective substrate recruitment must be encoded elsewhere.

Although PP1 by itself is not selective and can dephosphorylate a large number of proteins *in vitro*, PP1 holoenzymes are thought to be selective.^9,19^ Selectivity may be encoded by non-catalytic subunits through a range of non-mutually exclusive mechanisms including targeting to subcellular locations, altering substrate recognition properties, and direct binding to substrate.^9,19,20^

Knowledge of the molecular and structural determinants of substrate selectivity by PP1 holoenzymes is limited because many substrates are either large proteins or protein complexes and non-catalytic subunits are often intrinsically disordered proteins (IDPs). Such proteins represent a major challenge for structural studies.^21,22^ Elements of selectivity have emerged from a few pioneering studies using fragments of non-catalytic subunits either in the absence or presence of phospho-peptide substrates. These studies have revealed two modalities to explain how selectivity can be imparted by non-catalytic subunits: they can build a composite surface to produce an extended PP1 catalytic cleft^23^ and/or block access to non-cognate substrates.^24^

In addition to the above limitations, only a few PP1 holoenzymes have known substrates.^19^ Phosphorylation of serine 51 (S51) of the alpha subunit of heterotrimeric eukaryotic translation initiation factor 2 (eIF2α) is a vital signaling event regulated by the antagonistic actions of four kinases, each sensing different signals, and two holophosphatases.^19,25,26^ These eIF2α holophos-phatases are composed of PP1 bound to either PPP1R15A (R15A) or PPP1R15B (R15B). R15B is constitutively expressed, whereas R15A is stress induced.^19^ Structures of R15A^582–621^ and R15B^631–684^ have shown that they bind to PP1 through their RVxF motifs.^27,28^

Although the binding of non-catalytic subunits to PP1 is well understood, it is unknown how full substrates are recruited. It is also unclear whether holoenzymes are able to recruit substrates by themselves or whether additional factors are required. Thus, eIF2α holophosphatases provide a well-defined framework to study how PP1 holoenzymes recruit their substrates. We previously found that R15A and R15B contain a substrate-recruitment region that maps to 325–554 and 340–639, respectively.^29^ G-actin has also been proposed to confer selectivity to R15-PP1 complexes prepared with the C-terminal ~50 amino acids of R15s that harbor the RVxF motif.^27^ Although this C-terminal fragment of R15A binds PP1, it does not increase the enzymatic activity toward eIF2α,^27,30^ establishing that substrate recruitment involves an alternative mechanism. In a minimal reconstituted system, we have shown that the middle regions of R15A and R15B increase enzymatic activity,^30^ but the molecular determinants of this activity are unknown.

Understanding the molecular basis of substrate recruitment by eIF2α phosphatases is important in itself and can also serve as a paradigm for other holoenzymes. Human R15A and R15B are proteins of 674 and 713 residues, respectively, for which structural knowledge is confined to ~C-terminal 30 amino acids that encompass the PP1-binding motif.^27,28^ Because eIF2 is a trimeric complex of 1,120 residues, which is difficult to express and purify, all previous studies on eIF2α kinases and phosphatases have been done with the N-terminal fragment of eIF2α, encompassing S51.^28,31^

Here, we aimed at elucidating the mechanism by which R15B recruits its full 125 kDa trimeric eIF2 substrate. First, we defined a minimal substrate-binding domain of R15B (R15B^414–613^) and found that it bound with high affinity to eIF2 and purified the resulting complex. Due to the intrinsically disordered nature of the substrate-binding domain of R15B, we used a combination of approaches consisting of hydrogen/deuterium exchange mass spectrometry (HDX-MS), nuclear magnetic resonance (NMR) spectroscopy, and functional studies in mammalian cells to gain insights into the mechanism by which R15B recruits eIF2. HDX-MS analyses revealed a R15B binding region on the far end of the eIF2 complex surface relative to the phosphorylation site, a surprising finding as prior studies assumed that substrate recognition was mediated by the eIF2α N-terminal region, harboring the phosphorylation site. NMR analyses revealed that R15B^414–613^ is largely disordered, with the exception of three short and separate elements with α-helical propensity. AlphaFold2 also predicted these regions to be helical, hereafter referred to as H1 (424–429), H2 (472–482), and H3 (549–560). Both HDX-MS and NMR converged in showing that H1, H2, and, to a lesser extent, H3 contributed to binding eIF2. Mutagenesis studies in mammalian cells revealed that H1 is essential for R15B substrate-recruitment function and dephosphorylation activity. Establishing the importance of these findings for human health, a homozygous missense variant in the residue N-terminal to H1 (N423D) was identified in a patient with microcephaly, development delay, and intellectual disability. We found that the variant has reduced capacity for substrate recruitment and dephosphorylation, establishing the molecular mechanism of this rare condition. Our integrated experimental findings combined with an AlphaFold model explain how a large substrate can be captured by short α-helical elements within an IDP to efficiently promote its dephosphorylation.

## Results

### R15B^414–613^ binds 125 kDa human eIF2 with high affinity

**Figure 1:**
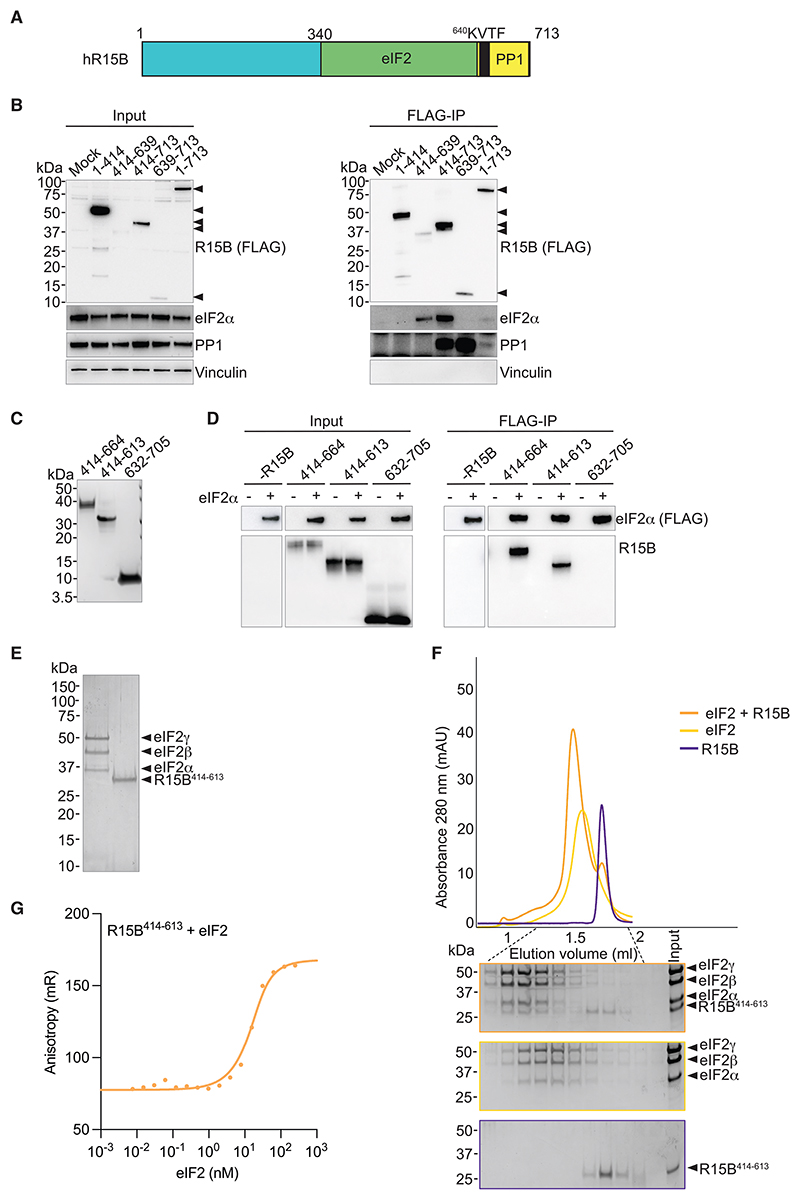
Reconstitution of a R15B-eIF2 complex with recombinant proteins (A) Cartoon illustrating the regions of R15B binding to eIF2 and PP1. (B) FLAG-R15B constructs were transfected into HEK293T cells (input) and immunoprecipitated using anti-FLAG M2 magnetic beads (FLAG-IP). Samples were eluted from the beads by boiling in LDS buffer and eluates were separated on a 4%–12% Bis-Tris Plus gel. Proteins were detected by immunoblotting with FLAG (black arrows), eIF2α, PP1, and vinculin antibodies. Representative results of at least 3 experiments are shown. (C) Coomassie-stained gel of purified R15B fragments. Molecular weight markers are indicated in kilodaltons (kDa). (D) FLAG immunoprecipitations from yeast lysates with overexpressed human eIF2, harboring a FLAG on eIF2α, with or without the indicated recombinantly expressed and purified R15B protein revealed by immunoblotting. (E) Coomassie-stained gel of purified human eIF2 (α, β, and γ) and R15B^414–613^. (F) Size exclusion chromatography (SEC) of R15B^414–613^, trimeric human eIF2 complex, and both combined. Bottom panel: SDS-PAGE of the SEC fractions. (G) Fluorescent anisotropy measurements with 20 nM NT-495-labeled R15B^414–613^ and titration of eIF2. Graph shows one representative replicate. Kd calculated from three replicates is 5.8 ± 0.94 nM standard error of mean.

In previous studies,^29,30^ we reported that the substrate-recruitment region of R15B is located between residues 340 and 639 (Figure 1A). This region is difficult to express recombinantly without a large solubilizing tag, a limitation that has hindered further progress. Sequence alignments revealed that the N-terminal ~70 amino acids of the substrate-recruitment region of R15B are less conserved (Figure S1), suggesting that they may be dispensable. Full-length R15B as well as R15B^414–713^ immunoprecipitated with both eIF2α and PP1, whereas R15B^414–639^ only immunoprecipitated eIF2α and R15B^639–713^ captured PP1, but not eIF2α (Figure 1B). R15B^1–414^ did not immunoprecipitate with either PP1 or eIF2α (Figure 1B). These results corroborate previous findings showing that substrate recognition and PP1 recruitment are encoded by non-overlapping regions of R15B.^29,30^ As previously reported, full-length R15B captured less eIF2α than fragments containing the substrate-binding region.^29^ Importantly, we show that the substrate-binding region of R15B is smaller than previously reported.^29^ This work establishes that residues 1–413 of R15B are dispensable for substrate recruitment.

We then aimed to define a recombinant system suitable for biochemical studies. R15B derivatives were expressed with a hexahistidine tag in *E. coli*, purified and tested for their ability to bind human eIF2 complex contained in yeast lysate^32^ (Figures 1C and 1D). We found that R15B^414–613^ and R15B^414–664^ were able to bind eIF2, whereas there was no detectable binding with R15B^632–705^ (Figure 1D). Recombinant R15B^414–613^ and trimeric eIF2 were purified (Figure 1E) and formed a complex that remained intact during gel filtration chromatography (Figure 1F), establishing that R15B^414–613^ binds directly to the trimeric substrate, without any additional components. We next used fluorescence anisotropy with labeled R15B and found that it interacted with eIF2 with a dissociation constant of 5.8 ± 0.94 nM (Figure 1G). Thus, we identified and used a fragment of R15B to reconstitute substrate recognition with the full trimeric eIF2 to demonstrate that R15B binds eIF2 with high affinity, without requiring PP1 or any other factor.

### R15B protects regions of the eIF2 complex opposite to the phosphorylation site

**Figure 2:**
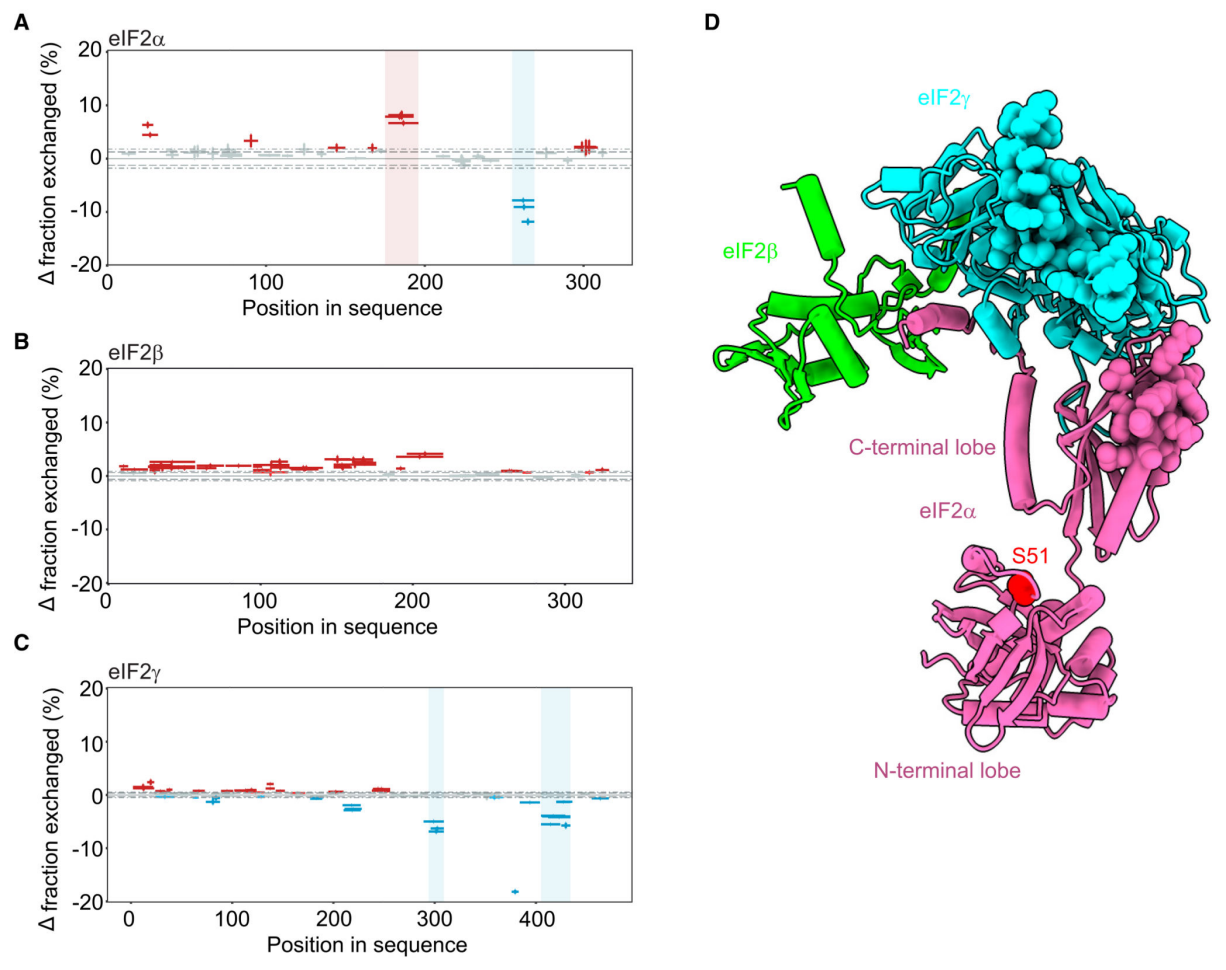
R15B binds to eIF2 in regions distant from the phosphorylation site (A–C) Woods plots showing differences in deuterium uptake for eIF2 alone or in the presence of R15B^414–613^. Deprotected, protected, and non-significantly different peptides are in red, blue, and gray, respectively, plotted as Δ fraction exchanged (y-axis). Bar length corresponds to peptide length plotted against the sequence position (x-axis). Dashed and dotted lines indicate 98% and 99% confidence intervals applied to identify peptides with statistically significant deuteration differences. Regions with significant differences (see STAR Methods) are highlighted in blue (increased protection) and red (decreased protection) rectangles. Representative time points are shown. (A) eIF2α, 5 min, (B) eIF2β, 3 s, and (C) eIF2γ, 3 s. Error bars denote combined uncertainty of peptide deuteration calculated based on triplicate experiments. (D) Mapping of the protected regions on an AlphaFold model of human eIF2 upon binding to R15B^414–613^. Protected residues are shown as spheres. No protection was observed on eIF2β, which is only partly resolved. The phosphorylation site, S51, is shown in red.

To gain structural insights on the R15B^414–613^-eIF2 complex, we performed HDX-MS. Although HDX-MS does not directly map protein-protein interactions, areas showing decreased hydrogen-deuterium exchange rates in the presence of a binding partner are likely to represent the binding site between the two proteins and areas with increased exchange indicate change in conformation.^33^ Purified eIF2 complex was mixed with D_2_O-containing buffer and incubated for varying times in the presence or absence of R15B^414–613^ (Figures 2A–2C and S2D–S2L; Table S1). Exchange was analyzed by mass spectrometry following proteolytic cleavage. HDX-MS analysis yielded high-sequence coverage with redundancy of peptides within eIF2 subunits (Figures S2A–S2C), and the most significant changes were analyzed (see STAR Methods). Surprisingly, differential HDX-MS analysis showed no protection in the N-terminal part of eIF2α around the phosphorylation site (Figure 2A). Increased deuterium exchange was observed for eIF2α in the connecting loop between the N- and C-terminal lobes (Figures 2A and 2D), suggesting a possible conformational change. Residues 255–269 in the C-terminal domain of eIF2α showed reduced deuterium uptake in the eIF2 complex in the presence of R15B^414–613^ (Figures 2A and S2D–S2F; Table S1). We did not observe a significant change in eIF2β upon binding to R15B (Figures 2B and S2G–S2I; Table S1). Unexpectedly, binding of R15B to eIF2 protected residues 294–309 and 405–436 at C-terminal end of eIF2γ (Figures 2C and S2J–S2L; Table S1). The regions with increased protection on eIF2 following the binding of R15B form a contiguous surface on the complex (Figure 2D). Thus, the reconstitution of R15B in complex with the full trimeric substrate reveals that R15B protects regions of the 125 kDa eIF2 complex remote from the phosphorylation site, particularly on eIF2γ and extending on the connecting C-terminal domain of eIF2α. These findings were unanticipated because prior work has focused on the N-terminal domain of eIF2α that harbors the phosphorylation site.

### R15B binds eIF2 through short and separated regions

**Figure 3:**
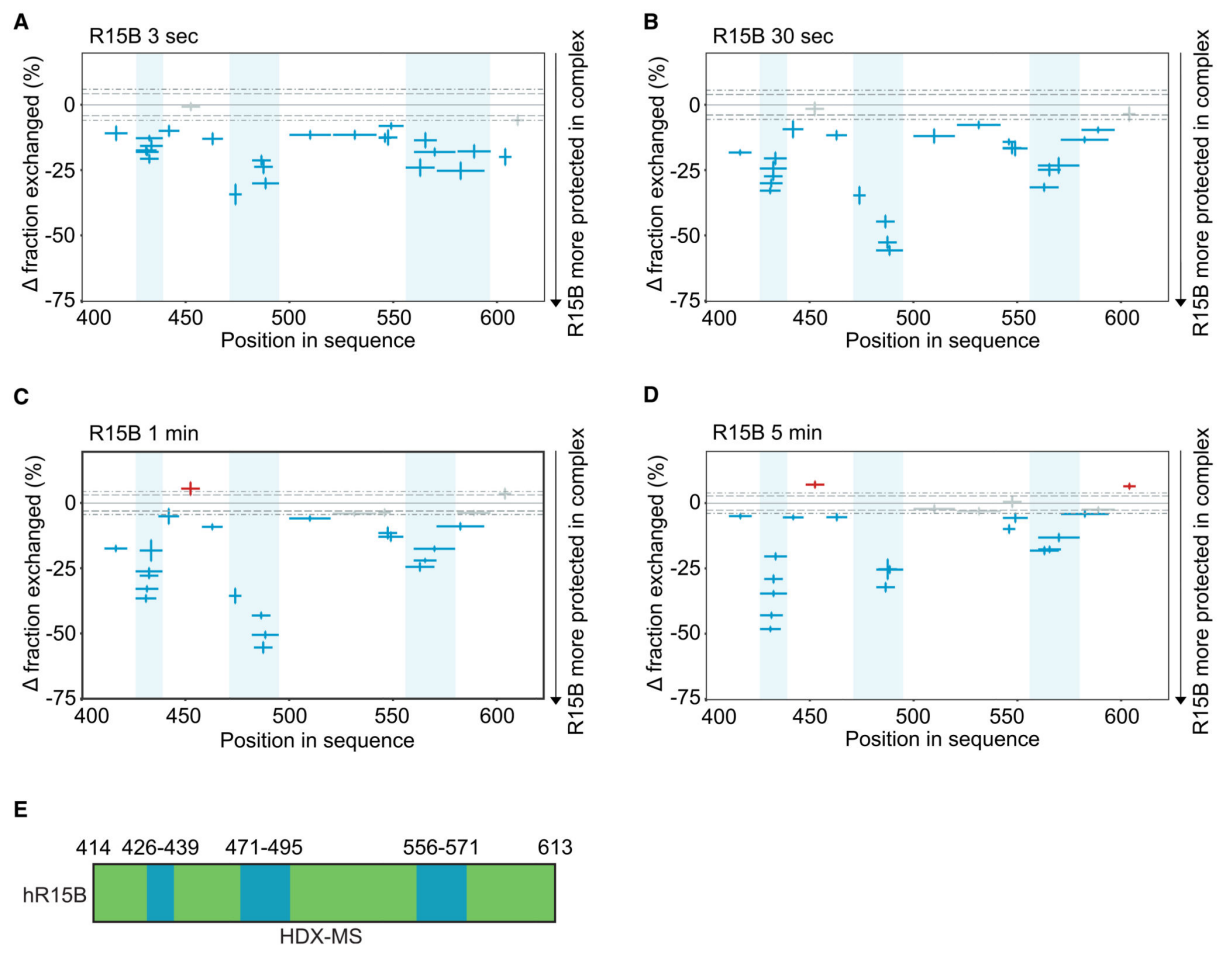
R15B binds eIF2 via discrete regions (A–D) Woods plots showing the difference indeuteration for a given R15B peptide at a given time point (A, 3 s; B, 30 s; C, 1 min; and D, 5 min) against the sequence position following addition of eIF2 complex. Deprotected, protected, and non-significantly different peptides are in red, blue, and gray, respectively, plotted as Δ fraction exchanged between the two states (y-axis). Bar length corresponds to peptide length plotted against the amino acid sequence (x-axis). Dashed and dotted lines indicate 98% and 99% confidence intervals applied to identify peptides with statistically significant deuteration differences. Regions with greatest differences are highlighted in blue. Error bars denote combined uncertainty of peptide deuteration calculated based on triplicate experiments. (E) Cartoon representation of the three short regions of R15B^414–613^ protected from deuteration upon binding to eIF2. Green depicts the substrate-recruitment module of R15B identified in Figure 1, and blue depicts the regions most protected upon binding to eIF2.

With these insights on the interactions between eIF2 and R15B, we next focused on R15B^414–613^ to learn how it binds its large substrate. IDPs are problematic to study for many reasons. In HDX, they exchange rapidly, as observed here for R15B with saturated deuterium uptake observed after 3 s in heavy water (Figure S3A). This rapid exchange is too fast to detect perturbations in HDX kinetics. To overcome this problem, we then turned to a method enabling expansion of the HDX time window by lowering the pH of the exchange reaction.^34^ The method was first validated with free R15B and showed a reduced rate of deuterium incorporation compared with standard conditions (Figure S3A). We next added an excess of eIF2 and analyzed the resulting perturbations on R15B over time. HDX-MS analysis yielded high-sequence coverage of R15B (Figure S3B). Addition of eIF2 resulted in increased protection throughout the sequence of R15B to various degrees (Figure 3; Table S1). The most pronounced changes localized to three short regions, 426–439, 471–495, and 556–571, with weaker changes in between (Figure 3). These HDX experiments suggest that the interaction with eIF2 occurs primarily through short and separated regions of R15B and that residues in between these regions, which exhibit weaker protection, may also contribute to the binding.

### R15B^414–613^ is intrinsically disordered

**Figure 4:**
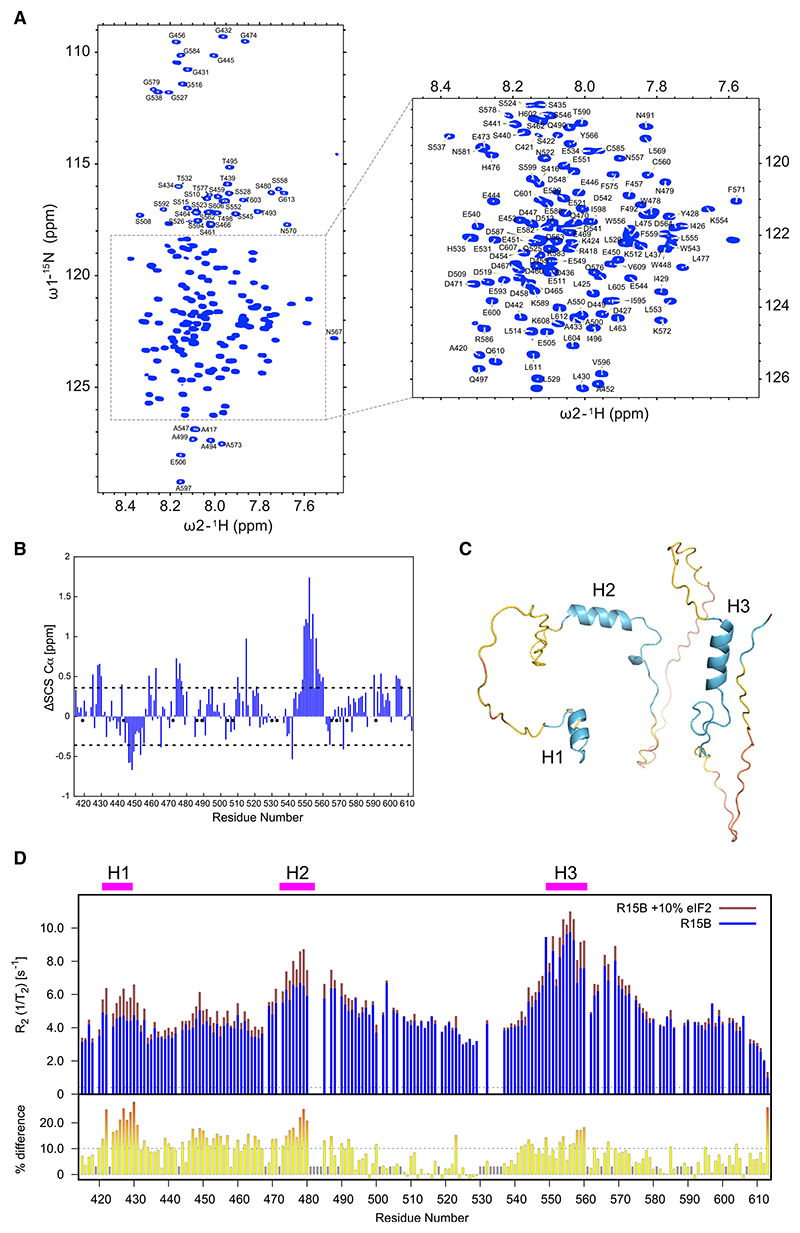
NMR reveals discrete helical elements of R15B that bind eIF2 (A) ^1^H,^15^N 2D HSQC of R15B^414–613^ with assignment of backbone amide resonances. The inset shows an expanded view of the central region. The narrow dispersion of ^1^H chemical shifts is a hallmark of intrinsically disordered proteins. (B) Secondary structure propensities of R15B^414–613^ characterized by the deviations of observed Cα chemical shifts from estimated random coil values (ΔSCS Cα). Negative values suggest a propensity for extended conformation, whereas positive deviations suggest increased likelihood of α-helical structure. Only consecutive stretches of residues with ΔSCS Cα values above or below 1 standard deviation are considered. (C) AlphaFold2 model of R15B^414–613^. The residue positions of predicted helices are 424–429 (H1), 472–482 (H2), and 549–560 (H3). The model is colored based on the predicted local distance difference tests (pLDDTs). Blue color in the model corresponds to a confident score (90 > pLDDT > 70), yellow to a low confidence score (70 > pLDDT > 50), and orange to a very low confidence score (pLDDT < 50). (D) Top panel: superposition of transverse relaxation R_2_ rates of R15B^414–613^ alone and in the presence of 10% eIF2. Bottom panel: observed transverse relaxation R_2_ rate differences. Significant changes above 1 standard deviation are highlighted in red.

To further investigate how R15B binds to trimeric eIF2, we used NMR spectroscopy.^35^ An NMR study has been previously reported with a C-terminal 70 amino acid fragment of R15A (R15A^552–621^),^17^ but there is no structural information on the substrate-recruiting region of R15B, which was predicted to be largely disordered by PSIPRED.^36^ Thus, we first conducted a detailed NMR analysis of R15B^414–613^ alone in solution. High-quality 2D HSQC spectra were obtained, yielding well-defined amide resonances that allowed the unambiguous assignment of 166 out of 186 non-proline residues (89%) (Figure 4A). The observed narrow dispersion in the ^1^H dimension (Figure 4A) indicates that R15B^414–613^ is predominantly intrinsically disordered in solution.

### Short secondary structure elements in R15B^414–613^ revealed by NMR

To investigate if R15B^414–613^ contains secondary structure elements, we calculated secondary chemical shifts as the difference between experimentally determined and theoretical random-coil Cα chemical shifts (Figure 4B). Stretches of significant positive secondary chemical shifts were observed for residues 428–430, 474–477, and 548–560, indicating α-helical propensity (Figure 4B). Negative secondary chemical shifts indicate an extended conformation for residues 446–449 (Figure 4B), possibly related to charge repulsions in this negatively charged region (Figure 4B). We next used AlphaFold2^37–39^ to model R15B^414–613^ (Figure 4C). In good agreement with the experimental data, AlphaFold2 predicted α-helices for residues 424–429, 472–482, and 549–560 (Figure 4C). We named these regions H1, H2, and H3, respectively (Figure 4C).

To gain further insights into the structural and dynamic properties of R15B^414–613^, we employed ^15^N backbone dynamics measurements. Heteronuclear Overhauser enhancements, ^15^N{^1^H} NOEs), sample predominantly fast, picosecond mobility, whereas longitudinal and transverse ^15^N-relaxation (R_1_ and R_2_) and ^15^N η_xy_-cross-correlated relaxation measurements provide insights into backbone mobility over a wider range of timescales. All relaxation measurements exhibit a similar sequence dependency with “peaks” and “troughs” along the sequence, with the exception of the R_1_ relaxation measurements (Figures S4A–S4D). Peaks in these dynamics measurements generally correlate with elements of increased backbone rigidity. Peaks in ^15^N{^1^H} NOE measurements (Figure S4A) aligned well with residues with helical propensity (Figure 4B). Similar profiles were observed for the R_2_ and η_xy_ data (Figures S4B and S4C). Together, our NMR data define R15B^414–613^ as a largely disordered protein with discrete regions with helical propensity.

### Discrete pre-formed helical elements in R15B^414–613^ bind to eIF2

Interaction studies by NMR frequently employ chemical shift perturbations. Although we obtained well-defined 2D HSQC spectra of R15B^414–613^ (Figure 4A), studying its binding to the 125 kDa eIF2 is challenging due to the high molecular weight of the R15B-eIF2 complex. Tightly bound residues are expected to experience a signal loss due to fast relaxation; only residues that retain a high degree of internal flexibility would remain observable. In addition, a combination of weak interactions via multiple binding sites may result in additional line broadening due to chemical exchange between different complexes.

To circumvent these issues and elucidate which regions of R15B are involved in binding its substrate, we studied the relaxation properties of R15B^414–613^ in the presence of a sub-stochiometric amount of eIF2. Incubation of R15B^414–613^ with 10% eIF2 resulted in localized peak intensity variations in R15B (Figure S4F), which confirmed that the addition of eIF2 altered the R15B conformational ensemble, a property we explored further. We observed few changes in ^15^N{^1^H} NOE or η_xy_ values upon addition of eIF2 (Figures 4G, S4A, S4C, and S4I). A small, general increase in the R_1_ values was observed upon addition of eIF2, probably as an indication of slower tumbling of the population-averaged ensemble (Figures S4D and S4J). A striking increase in the transverse relaxation rates, R_2_, was observed for regions encompassing the previously defined α-helical elements of R15B^414–613^ (Figures 4D, S4B, and S4H). The sensitivity of these R_2_ measurements upon sub-stochiometric addition of eIF2 is explained by the large molecular weight difference between free and eIF2-bound R15B.

To estimate the additional contribution of conformational exchange between the free and bound state in the R_2_ measurements, we calculated “exchange-free” transverse relaxation R_2_ rates according to Rezaei-Ghaleh et al.^40^ The previously defined helical elements show elevated differences between the measured and calculated exchange-free R_2_ rates, highlighting relaxation through increased conformational exchange upon eIF2 addition in these regions (Figures S4E and S4K).

Together, these NMR analyses revealed 3 sites in R15B that undergo binding-induced dynamic changes: those were most significant for residues 421–431, followed by residues 474–480 and, to a lesser extent, 558–560 (Figure 4D) and coincided with elements of R15B that adopt an α-helical conformation (Figure 4B). Less pronounced, but also significant, changes were observed between the first 2 helical binding elements (Figure 4D), suggesting that this extended region might contribute to the binding. Thus, we propose that R15B interacts with eIF2 using the pre-formed helical elements H1, H2, and, to a lesser extent, H3, with a potential involvement of residues between H1 and H2. These findings converge with the HDX analyses.

### An R15B mutant defective in substrate recruitment and catalysis

**Figure 5:**
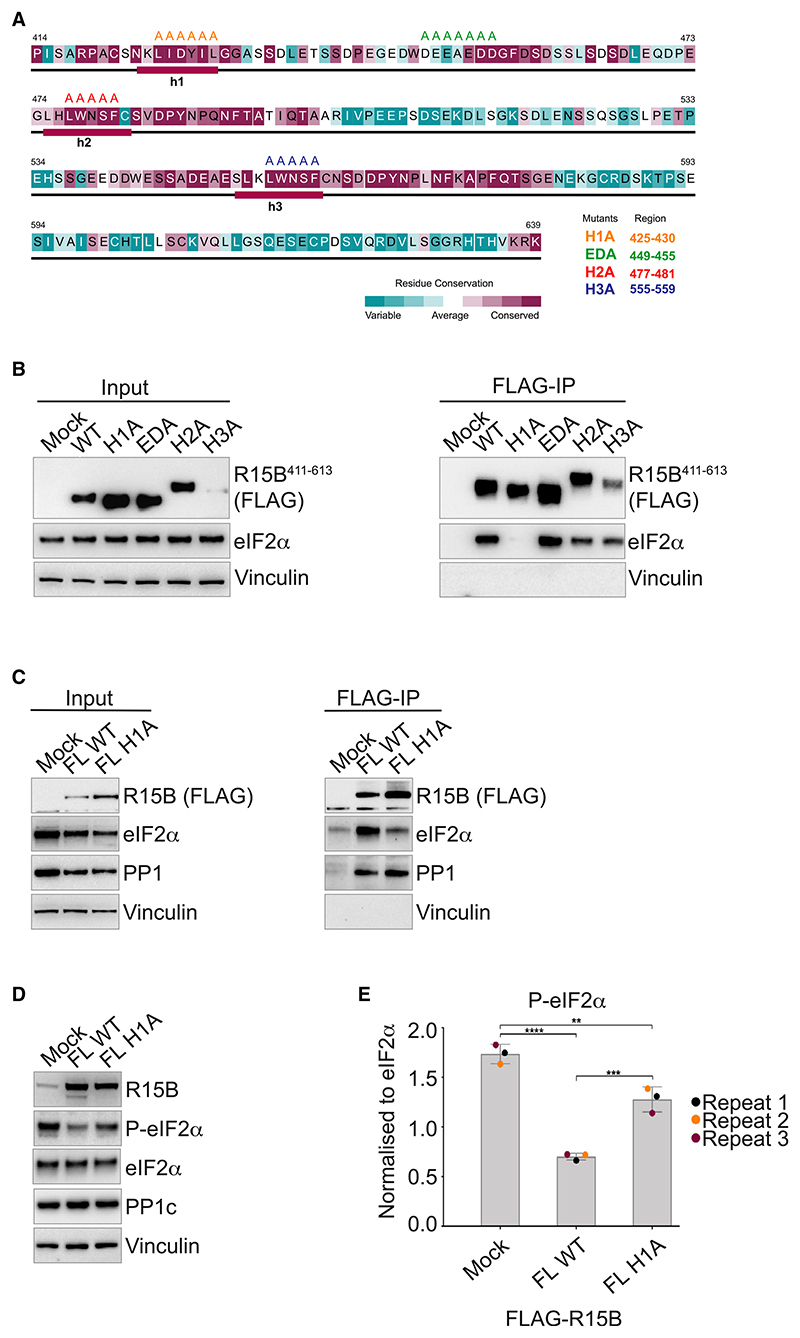
Identification of a mutant of R15B defective in substrate binding (A) Sequence conservation of R15B^414–639^. The residues are colored according to ConSurf^41^ conservation scores from cyan (variable) to burgundy (conserved). The consensus secondary structure, predicted using Jpred^42^ and PsiPred,^36^ is shown below the corresponding sequence. The secondary structural elements are denoted as follows: rectangle, helix; line, random coil. The substitutions of the residues targeted for mutagenesis are shown on the top with different mutants shown in different colors. (B and C) R15B^411–613^ (B) or full-length (C) wild-type (WT) or mutants were transfected into HEK 293T cells (input) and immunoprecipitated using anti-FLAG M2 magnetic beads (FLAG-IP). Immunoprecipitated complexes were eluted and analyzed on a 4%–12% bis Tris Plus gel. Proteins were detected by immunoblotting with FLAG, eIF2α, PP1, and vinculin antibodies. Representative results of at least 3 experiments are shown. (D) Activity of transfected R15B full-length WT or H1A assessed by decreased levels of P-eIF2α. Proteins were detected by immunoblotting with R15B (R15B-4D11), P-eIF2α, eIF2α, PP1, and vinculin antibodies. Representative results of at least 3 experiments are shown. (E) Quantifications of P-eIF2α from 3 experiments such as the one shown in (D). Data are mean ± SD. (n = 3). **p < 0.01, ***p < 0.001, ****p < 0.0001, as determined by one-way ANOVA.

In parallel to structural approaches, we also aimed at identifying the substrate-binding region through functional studies in human cells. Sequence conservation analysis of R15B^414–63941^ and consensus secondary structure prediction^36,42^ (Figure 5A) revealed that this part of the protein is largely disordered with the exception of three conserved helical regions, H1, H2, and H3, previously identified (Figures 4B and 4C). The analysis also revealed that R15B^414–639^ is composed of two repeats 414–498 and 499–577 (Figure S5A). The conservation within these repeats (Figure S5A) correlates quite well with the conservation across species (Figure 5A), suggesting that they are probably evolutionarily related. However, their N-terminal parts are less conserved (Figure S5A). In the second repeat of R15B, the region corresponding to H1 in the first repeat has a different amino acid composition and, consistent with this divergence, is not predicted to be helical (Figure 5A). Interestingly, the N-terminal part of the first repeat of R15B^414–639^ revealed similarity to three of the four repeats identified in R15A (Figure S5B). The four repeats of R15A have been previously outlined,^43^ and the three N-terminal repeats have been reported to bind eIF2α.^17,44^ Based on these sequence analyses, four regions in R15B were targeted for mutagenesis: H1 that is unique to R15B repeat 1 and has some similarity to regions in R15A (Figure S5B), the ED motif enriched with acidic residues that is conserved in R15B across species as well as in the R15A repeats, and H2 and H3 that are conserved in both R15B repeats (Figure 5A). H1, H2, H3, and the ED motifs were replaced by alanines, generating H1A, H2A, H3A, and EDA mutants. The mutants expressed at levels similar to the wild type (WT), except H3 (Figure 5B). eIF2α co-precipitated with R15B^411–613^ WT as well as EDA, H2A, and H3A (Figure 5B). R15B^411–613^ with the H1A mutation was immunoprecipitated at levels similar to the WT proteins, but the capture of eIF2α was dramatically reduced (Figure 5B). H2A was slightly compromised, whereas H3A seemed more efficient at capturing the substrate when compared to WT (Figure 5B). When implemented in the full-length protein, the H1A mutation also markedly compromised substrate binding without altering PP1 binding (Figure 5C). Of note, H1 is not only common to R15A and B but also conserved in the *Drosophila* R15 as well as the viral R15-related proteins, ICP34 and DP71L (Figure S5C).

To assess the functional importance of H1 for catalysis, we compared the ability of overexpressed WT full-length R15B to dephosphorylate eIF2α with that of the H1A mutant. We found that the activity of R15B^H1A^ was compromised (Figures 5D and 5E). Importantly, H1 encompasses the region of R15 showing high perturbations upon binding to eIF2 in the HDX-MS (Figure 3) and NMR studies (Figure 4D). H2 was also perturbed in the NMR experiments, albeit less than H1 (Figure 4D). Thus, we propose that H1 serves to anchor R15B to eIF2 and H2 contributes an additional binding site.

### A genetic variant in the substrate recognition module of R15B reduces its function and causes microcephaly, developmental delay, and intellectual disability

**Figure 6:**
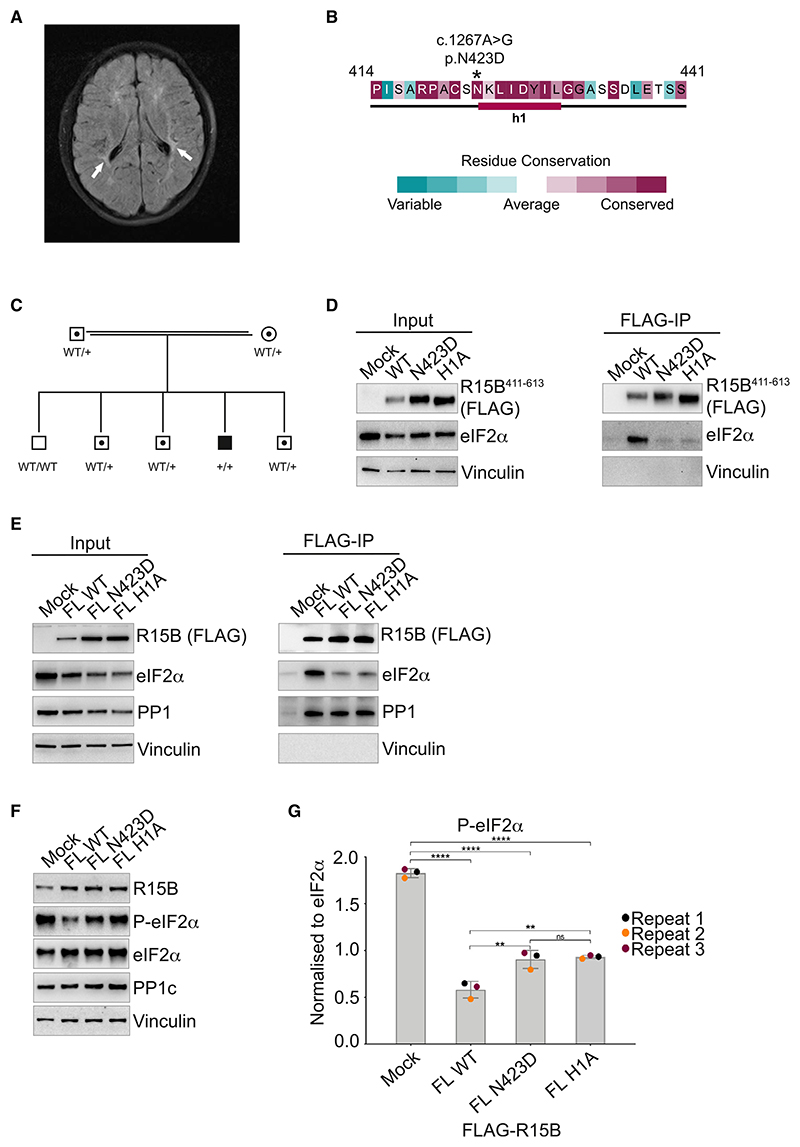
A homozygous missense variant in the substrate recognition module of R15B causes microcephaly, developmental delay, and intellectual disability (A) Magnetic resonance imaging (fluid-attenuated inversion recovery [FLAIR] sequence) of the brain of the R15B^N423D^ boy showing periventricular white matter hyperintensities (white arrows). (B) A homozygous variant c.1267A>G in *PPP1R15B* translates in a N423D missense in R15B’s substrate recognition module in a boy with microcephaly. (C) Pedigree of the family with the c.1267A>G in *PPP1R15B*. Circles refer to females, squares refer to males. An enclosed dot represents heterozygosity and a solid fill represents homozygosity for the c.1267 A>G PPP1R15B variant. Wild type (WT); +: variant c.1267 A>G allele. (D) R15B^411–613^ (E) or R15B full-length (FL) wild-type (WT), N423D or H1A mutants were transfected into HEK293T cells (input) and immunoprecipitated using anti-FLAG M2 magnetic beads (FLAG-IP). Immunoprecipitated complexes were eluted and analyzed on a 4%–12% bis Tris Plus gel. Proteins were detected by immunoblotting with FLAG, eIF2α, PP1, and vinculin antibodies. Representative results of at least 3 experiments are shown. (F) Activity of transfected R15B FL WT, R15B FL N423D, and R15B FL H1A assessed by decreased levels of P-eIF2α. Proteins were detected by immunoblotting with R15B (R15B-4D11 in house), P-eIF2α, eIF2α, PP1, and vinculin antibodies. Representative results of at least 3 experiments are shown. (G) Quantifications of P-eIF2α from 3 experiments such as the one shown in (E). Data are mean ± SD. (n = 3). **p < 0.01, ****p < 0.0001, ns, not significant, as determined by one-way ANOVA.

While this experimental work was being finalized, a rare variant in R15B associated with a human syndrome was discovered in a 9-year-old boy with global developmental delay and intellectual disability (IQ 57). Physical examination showed a short stature (–2.16 SD), overweight (weight to length ratio at +3.49 SD), microcephaly (–2.60 SD), hypermobility, and thin lower legs. Mild dysmorphic features were present: a round face with full cheeks, upslant palpebral fissures, and a flat midface. Gross neurological examination was unremarkable except for brisk reflexes of the lower extremities. Magnetic resonance imaging of the brain showed periventricular white matter hyperintensities (Figure 6A). Single-nucleotide polymorphism (SNP) array revealed no deletions or duplications. However, several regions of homozygosity were present, consistent with the consanguinity of the parents. Whole exome sequencing identified a homozygous missense variant in the PPP1R15B gene, c.1267A>G resulting in a N423D mutation in the protein (Figure 6B). Both parents are consanguineous heterozygous carriers, and segregation analysis excluded homozygosity in the four healthy siblings (Figure 6C). Additional laboratory testing in the patient showed normal glucose (5.3 mmol/l), HbA1c (34 mmol/mol), ALAT (18 U/l), ASAT (28 U/l), and gamma GT (11 U/l) levels. Abdominal sonography was normal.

The N423D variant in the patient lies at the N terminus of H1 (Figure 6B), a residue showing perturbation upon binding to eIF2 by NMR (Figure 4D). To test the consequence of the N423D variant, we introduced it in R15B^411–613^ as well as the full-length R15B and tested the properties of the mutant proteins in cell-based assays. When overexpressed in HEK293T cells, both H1A and N423D mutants captured significantly less eIF2α than the WT R15B^411–613^ fragment or full-length protein (Figures 6D and 6E). PP1 capture was tested with the full-length proteins because R15B^411–613^ does not bind PP1 (Figures 1A and 1B).^29^ Both N423D and H1A mutants captured PP1 at levels comparable with the WT R15B (Figure 6E), consistent with the fact that the mutations are outside of the PP1-binding region.^29^ Thus, the N423D variant identified in this human syndrome reduced substrate binding without impacting PP1 binding. This reduced eIF2α binding in the N423D and H1A mutants resulted in decreased, but not abolished, dephosphorylation activity to-ward P-eIF2α in cell lysates when compared with the WT R15B (Figures 6F and 6G). Thus, this identifies a disease-associated variant in R15B substrate recognition module that reduced its ability to recruit and dephosphorylate the substrate eIF2α.

### An AlphaFold model integrates key findings and explains substrate recruitment by R15B

**Figure 7:**
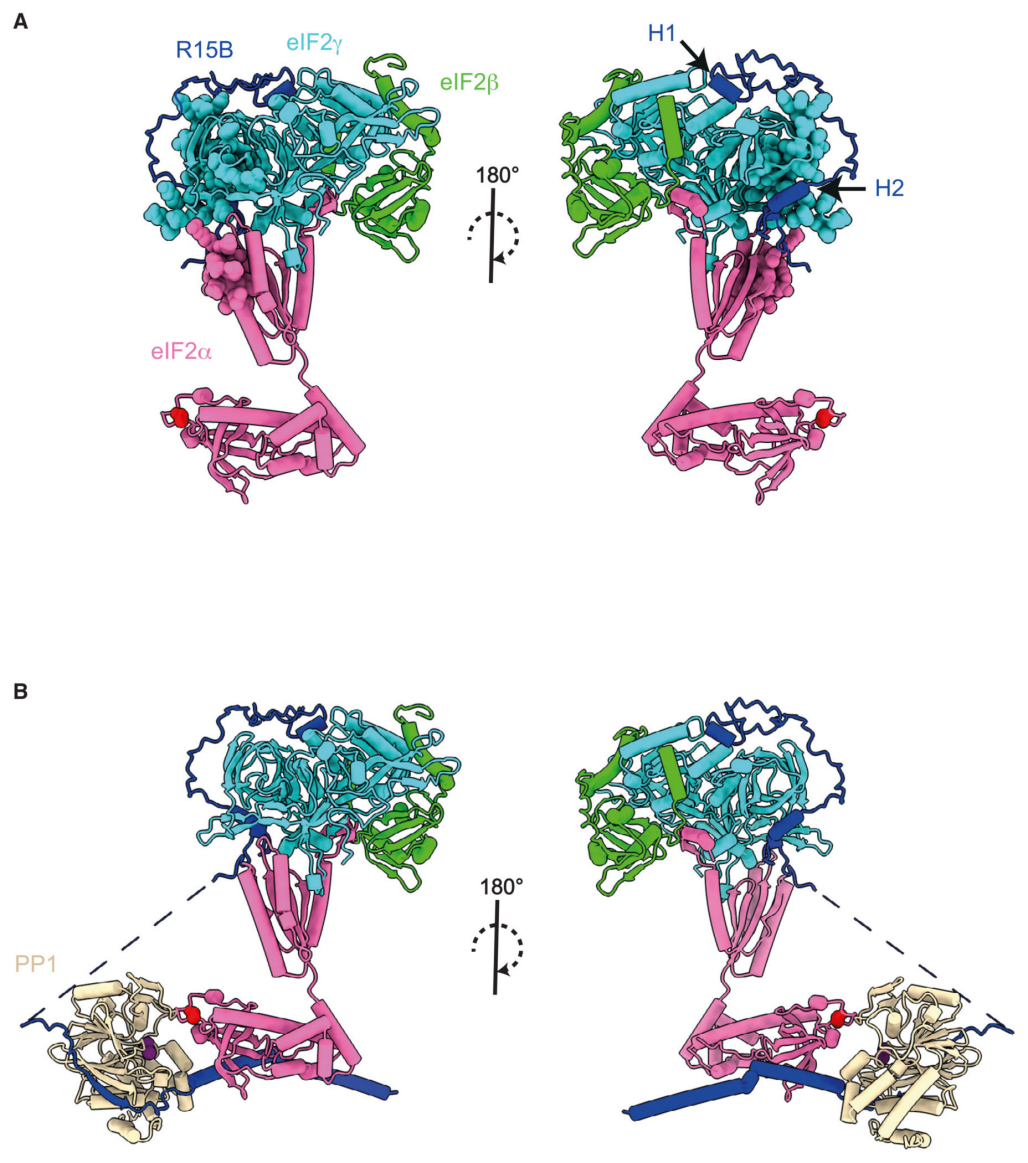
Model of recruitment of trimeric eIF2 by R15B (A) AlphaFold2 model of eIF2-R15B complex generated using the sequences of eIF2α and eIF2γ full-length, eIF2β^167–333^ and R15B^414–500^ as input. R15B^501–613^ and N and C termini of eIF2β were excluded from the model because of pLDDT scores <50. Residues on eIF2 that show significant change in deuterium uptake upon binding to R15B are shown as spheres. The phosphorylation site, S51 of eIF2α, is shown as a red sphere. (B) Composite model of (A) and an AlphaFold2 model of PP1-R15B^637–713^ with the N terminus of eIF2α. The sequence N-terminal to the R15B^636–713^ is shown with a dashed line. Metal ions (purple) were added to PP1 active site by using PDB: 3E7A as a template.

We next used AlphaFold2 to model the eIF2-R15B^414–613^ complex. The resulting model has high confidence scores for trimeric eIF2 and parts of R15B^414–500^ (Figure 7A). No confident structure predictions were obtained for residues 501–613 of R15B, which were thus removed from the final model (Figures S6A and S6B). The disordered N and C termini of eIF2β were also omitted in the model (Figure 7A). In the model, the predicted structure was similar to a cryoelectron microscopy (cryo-EM) structure of eIF2 in the ribosome pre-initiation complex (PDB: 6ZMW),^45^ with eIF2γ providing the core of the complex, eIF2β docked on one side, and eIF2α attached on the other (Figure 7A). eIF2α is bound to eIF2γ via its C terminus, whereas the flexible N terminus, harboring S51, is free (Figure 7A), as previously described.^46,47^ H1 of R15B binds at the interface between the G-domain and DIII of eIF2γ (Figure 7A). The region between H1 and H2 of R15B forms a loop that wraps around the surface of DIII of eIF2γ (Figure 7A). H2 binds near the interface between eIF2γ and the C terminus of eIF2α, with the region downstream of H2 proceeding toward eIF2α C-terminal domain (Figure 7A). Supporting the model, H1 and H2 of R15B were found to be protected from deuterium uptake upon binding to eIF2 (Figure 3). The NMR experiments also showed that R15B interacts with eIF2 using pre-formed helical elements H1 and H2 with potential involvement of the region in between (Figure 4D). The regions of eIF2 showing reduced deuterium uptake in the presence of R15B^414–613^ (Figure 2) are in close proximity to R15B (Figure 7A). Thus, the combination of HDX-MS, NMR, and AlphaFold modeling converge to reveal how R15B recruits eIF2, independent of PP1 binding.

We have previously reported that substrate recognition and PP1 recruitment are encoded by non-overlapping regions of R15B.^29,30^ To model the holoenzyme, we next generated an AlphaFold2 model of a PP1-recruiting fragment of R15B (R15B^637–713^) with PP1 and the N-terminal of eIF2α and merged the resulting model with the previous one (Figure 7B). This composite model of the holophosphatase bound to its trimeric substrate reveals how R15B grabs eIF2 in a region remote from S51 of eIF2α. In this way, R15B enables accurate presentation of S51 to the catalytic site of PP1 for effective catalysis.

## Discussion

Here, we present a combination of independent approaches that converge in revealing how a non-catalytic subunit of PP1 captures its full 125 kDa heterotrimeric substrate. Although IDPs represent a challenge for structural biology, combining HDX-MS, NMR, and mutagenesis studies in mammalian cells reveals how the substrate-recruitment region of R15B captures the full trimeric eIF2. We found that R15B binds eIF2 with high affinity, without requiring PP1 or any other factors. NMR revealed that R15B is largely disordered with short pre-formed helical elements. These regions are part of H1 (424–429), H2 (472–482), and H3 (549–560) helices predicted by AlphaFold. NMR perturbation experiments performed in the presence of eIF2 combined with mutagenesis studies show that R15B binds to eIF2 primarily through H1 and H2. These findings were corroborated using an HDX-MS method tailored to study IDPs. NMR also shows some perturbations in the region between H1 and H2, indicating that this region is likely to make contact with eIF2, as visualized in the AlphaFold model. Thus, independent lines of investigation reveal that H1, H2, and the disordered region between these helices form a clamp between helices H1 and H2 to grasp the substrate in a region remote to the phosphorylated residue. The essential H1 region is likely to anchor R15B to the substrate with other regions, including H2 contributing to binding. Importantly, H1 is not only common to R15A and B but also conserved in the *Drosophila* R15 as well as the related viral ICP34 and DP71L. The distal contacts allow R15B to firmly grasp the large substrate while avoiding steric clashes with the phosphorylated residue that needs to be accessible to the catalytic subunit PP1 for dephosphorylation. R15B contacts both the substrate and the catalytic subunit in sites remote from S51 and the active site, respectively, to position the two in close proximity for effective dephosphorylation.

The finding of an autonomous substrate-recruitment module demonstrates that the substrate can bind R15 in the absence of PP1 or any other factor. Although this work focused on the isolated substrate recognition module, our findings raise the possibility that the substrate could assemble with R15 independently or prior to PP1 binding, suggesting a two-step pathway for catalysis, where substrate recruitment by a module distant from the catalytic subunit precedes accurate and efficient delivery of the phosphorylation site to the catalytic subunit.

This work primarily focuses on R15B, and however, HDX-MS experiments provided insights into how R15B binds to a surface on eIF2 containing eIF2γ as well as the C-terminal of eIF2α. This changes the way one thinks about eIF2 recruitment. Prior structural work so far, with eIF2α kinases and phosphatases,^28,31^ was conducted with only the N terminus of eIF2α that harbors the phosphorylation site because it was anticipated that this fragment was sufficient for substrate recognition and catalysis. Working with the 125 kDa substrate presented challenges, but our findings reveal a recruitment mechanism that could not be elucidated with isolated fragments. This is an important lesson of broad relevance for future studies aiming at understanding phosphatase function. We found that R15B captures eIF2 by binding to a region on the far end of the complex relative to the phosphorylation site, opening up a new modality in substrate recognition by PP1 holoenzymes.

By a remarkable coincidence, a missense variant causing a rare human syndrome was found in a residue at the start of H1, providing human pathological relevance to the mechanism by which R15B recognizes its substrate. Although only one individual has been identified so far with the N423D variant, we propose that it is causative of the syndrome. The clinical picture, including short stature, microcephaly, and intellectual disability, is similar to previously reported patients with a R658C variant, reducing PP1 binding.^48,49^ We show that the N423D variant dramatically reduces substrate recruitment and dephosphorylation, demonstrating that this variant is hypomorphic. Thus, the similar clinical features of the R658C and N423D syndromes are explained by the loss of R15B function, although the underlying mechanism is different. The N423D variant has no effect on PP1 binding but decreases substrate recruitment and dephosphorylating activity, whereas the R658C reduces dephosphorylation by decreasing PP1 binding. These findings reveal, in humans, the importance of the previously uncharacterized substrate-recruitment function of R15B.

Substrate specificities of protein kinases are also often determined, at least in part, by substrate-binding regions, also called docking sites, distant to the phospho-acceptor site.^50^ A crystal structure of the NTD-eIF2α in complex with PKR has revealed the existence of a docking site peripheral to the phosphorylation site.^31^ Indeed, peptides containing the phosphoacceptor sites without the docking site are phosphorylated with 1,000-fold less efficiency than full-length protein eIF2α.^51^ Moreover, replacement of S51 of eIF2α by tyrosine still allows efficient phosphorylation by PKR.^31,52^ Thus, the modular organization of substrate recruitment and catalysis may be a principle shared by kinases and phosphatases, with the catalytic site having relaxed specificity and selectivity being ensured by independent modules. In light of the findings presented here, one wonders if eIF2α kinases engage their substrate with additional docking sites on eIF2γ and/or eIF2β.

eIF2α phosphorylation is an emerging therapeutic target for a broad range of diseases, from cancer to neurodegeneration.^53,54^ In previous work, we discovered inhibitors of R15A (Guanabenz and Sephin1) and R15B (Raphin1).^30,55–57^ Guanabenz and Sephin1 have recently entered the clinic with Guanabenz showing efficacy in a phase 2 clinical trial for ALS,^58^ and Sephin1 has successfully gone through a phase 1 clinical trial (https://clinicaltrials.gov/ct2/show/NCT03610334). The discovery of the molecular basis of substrate recruitment reported here offers an opportunity to design additional inhibitors to block the recruitment of eIF2α phosphatases and expand the range of compounds for studying the benefit of prolonging eIF2α phosphorylation experimentally and clinically. Targeting the substrate recognition module will provide selectivity, which cannot be achieved through targeting the PP1-binding motifs or PP1.

There are only a few PP1 holoenzymes for which substrates are known. Structural insights for PP1 non-catalytic subunits bound to substrates are scarce and were generated with peptides or fragments of the substrates, revealing contacts between the non-catalytic subunit and the peptide substrate in the proximity to the phosphosite^23^ Although such proximal contacts are likely to be important to stabilize the substrate near the active site of PP1, we propose that substrate recruitment by a module distant from the phospho-site followed by delivery of the phosphorylated residue to PP1 may be relevant for other PP1 non-catalytic subunits. For example, the C-terminal 291 residues of the MYPT1 subunit are known to directly bind myosin.^59^ There is no structural information on this domain that is likely to contribute to catalysis by recruiting the substrate. In contrast to the C-terminal domains of R15, which do not alter the activity of PP1 to the N-terminal fragment of eIF2α,^27,30^ the PP1 binding domain of Phactr1 (Phactr1^516–542^) increases catalysis 100-fold by extending the catalytic grove of PP1 and providing a selective docking site for phosphopeptide substrates.^23^ However, the reported k_M_ of Phactr1^516–542^-PP1 for the peptidic substrates, comprised between 33 and 704 μM, suggests that additional substrate-recruitment sites ought to exist to allow efficient and accurate capture of the substrate in cells.

Here, we explain how a large substrate can be captured on a site remote from the phosphorylation site by short helical elements in an IDP to present 1 residue to the catalytic phosphatase and efficiently promote its dephosphorylation, a function essential for human health. The work presented here using R15B and its full substrate yielded the discovery of the recruitment mechanism, a paradigm of broad relevance.

### Limitations of the study

In this study, we identified and used a fragment of R15B to reconstitute substrate recognition with the full trimeric eIF2 and elucidate how R15B binds eIF2. Although this is the largest fragment of R15B studied so far biochemically, it is not a full-length protein, and the next challenge will be to reconstitute complexes with the full R15B and PP1. Despite this limitation, the biological relevance of the findings was validated in cells and further demonstrated by a variant reducing substrate binding and dephosphorylation in a rare syndrome with microcephaly, development delay, and intellectual disability.

## Star⋆Methods

### Key Resources Table

**Table:** 

REAGENT or RESOURCE	SOURCE	IDENTIFIER
Antibodies		
Anti-PP1 (E-9) antibody	Santa Cruz	sc-7482; RRID: AB_628177
Anti-eIF2S1 antibody	Abcam	ab26197; RRID: AB_2096478
Recombinant Anti-eIF2S1 (phospho S51) antibody (E-90)	Abcam	ab32157; RRID: AB_732117
Anti-Vinculin antibody	Cell Signaling Technology	4650S; RRID: AB_10559207
Anti-FLAG antibody	Sigma-Aldrich	F7425; RRID: AB_439687
Anti-R15B-3E11	This paper	N/A
Anti-PPP1R15B antibody	Proteintech	14634-1-AP; RRID: 2300036
Anti-Rabbit IgG (H+L), HRP conjugate secondary antibody	Promega	W4011; RRID: 430833
Anti-Mouse IgG (H+L), HRP conjugate secondary antibody	Promega	W4021; RRID: 430834
Bacterial and virus strains		
One Shot BL21 Star (DE3) chemically competent *E.coli*	Thermo Fisher Scientific	C601003
Chemicals, peptides, and recombinant proteins		
cOmplete EDTA-free Protease Inhibitor Cocktail	Sigma-Aldrich	11873580001
cOmplete, Mini, EDTA-free protease Inhibitor Cocktail	Roche	04639159001
Lysozyme	Sigma-Aldrich	L6876
Benzonase nuclease	Sigma-Aldrich	E1014
Ni-NTA agarose	Qiagen	30210
PreScission protease GST-3C	Cytiva	GE27-0843-01
Aprotinin	Sigma-Aldrich	10236624001
Leupeptin	Sigma-Aldrich	L2884
Pepstatin A	Sigma-Aldrich	P5318
Anti-FLAG M2 magnetic beads	Sigma-Aldrich	M8823
3X FLAG peptide	Sigma-Aldrich	F4799
EIF2AK2 (PKR) recombinant human protein GST-tagged	Thermo Fisher Scientific	PV4821
PEI MAX transfection grade linear polyethylenimine Hydrochloride	Polysciences	24765
Ponceau S solution	Sigma-Aldrich	P7170
ECL Prime Western Blotting System	Cytiva	RPN2232
Deuterium oxide 0.75 ml ampoules	Thermo Fisher Scientific	351430075
Deuterium oxide (99.96% D) for NMR spectroscopy	VWR Chemicals	87156.0100
Deuterium chloride solution	Sigma-Aldrich	543047-50G
Sodium deuteroxide solution	Sigma-Aldrich	176788-50G
Riboflavin	Sigma-Aldrich	R9504
Niacinamide	Sigma-Aldrich	N5535
Pyridoxine hydrochloride	Sigma-Aldrich	P9755
Thiamin hydrochloride	Sigma-Aldrich	T1270
Disuccinimidyl dibutyric urea (DSBU)	Thermo Fisher Scientific	A35459
Iodoacetamide	Sigma-Aldrich	I1149-5g
Modified trypsin, sequencing grade	Promega	V5111
Critical commercial assays		
QuickChange II site-directed mutagenesis kit	Agilent	200523
Protein labeling Kit BLUE-NHS monolith (amine reactive)	Nanotemper	MO-L003
Agilent sureselect target enrichment Clinical Research Exome V2	Agilent	5190-9500
Deposited data		
NMR datasets are deposited in BMRB with accession code.	This paper	BMRB: 52233
The full HDX-MS datasets are included as Supplementary datasets and are deposited in PRIDE	This paper	PRIDE: PXD047538
Uncropped western blot images	This paper	Mendeley Data: https://doi.org/10.17632/v26bzg9833.1
Experimental models: Cell lines		
Human embryonic kidney 293T cells	ATCC	CRL-3216
Experimental models: Organisms/strains		
*S. cerevisiae:* Strain background: GP6452	Graham Pavitt	N/A
Oligonucleotides		
See Table S2	Sigma-Aldrich	N/A
Recombinant DNA		
R15B^1-414^ PXJ41 (N-FLAG)	This paper	N/A
R15B^411-613^ PXJ41 (N-FLAG)	This paper	N/A
R15B^414-639^ PXJ41 (N-FLAG)	This paper	N/A
R15B^414-713^ PXJ41 (N-FLAG)	This paper	N/A
R15B^639-713^ PXJ41 (N-FLAG)	Hodgson et al.^29^	N/A
R15B^1-713^ (Full-length) PXJ41 (N-FLAG)	Hodgson et al.^29^	N/A
H1A R15B^414-613^ PXJ41 (N-FLAG) residues 425-430 AAAAA	This paper	N/A
EDA R15B^414-613^ PXJ41 (N-FLAG) residues 449-455 AAAAAA	This paper	N/A
H2A R15B^414-613^ PXJ41 (N-FLAG) residues 477-481 AAAAA	This paper	N/A
H3A R15B^414-613^ PXJ41 (N-FLAG) residues 565-569 AAAAA	This paper	N/A
H1A R15B^1-713^ PXJ41 (N-FLAG) residues 425-430 AAAAA	This paper	N/A
N423D R15B^411-613^ PXJ41 (N-FLAG)	This paper	N/A
N423D R15B^1-713^ PXJ41 (N-FLAG)	This paper	N/A
R15B^414-613^ pET-47b (N-His-3C)	This paper	N/A
R15B^414-664^ pET-47b (N-His-3C)	This paper	N/A
R15B^632-705^ pET-47b (N-His-3C)	This paper	N/A
eIF2α pEBMulti-Neo	Riken BRC DNA Bank	RDB17725
eIF2β pEBMulti-Neo	Riken BRC DNA Bank	RDB17726
eIF2γ pEBMulti-Neo (N-FLAG-His)	Riken BRC DNA Bank	RDB17727
CDC123 pEBMulti-Neo	Riken BRC DNA Bank	RDB17728
Software and algorithms		
ProteinLynx Global Server	Waters	www.waters.com/waters/en_US/ ProteinLynx-Global-SERVER-(PLGS) /nav.htm?cid=513821&locale=en_US; RRID: SCR_016664
DynamX software	Waters	https://www.waters.com/waters/library.htm?locale=en_US&lid=134832928
NMRPipe	Jaravine et al.^60^	https://www.ibbr.umd.edu/nmrpipe/install.html
MddNMR package	Jaravine et al.^60^	http://mddnmr.spektrino.com
Topspin 3.6.0	Bruker	https://www.bruker.com/en/products-and-solutions/mr/nmr-software/topspin.html;RRID: SCR_014227
NMRFAM-Sparky 1.47	Lee et al.^61^	https://nmrfam.wisc.edu/nmrfam-sparky-distribution/;RRID: SCR_014228
Mars	Jung and Zweckstetter^62^	http://www3.mpibpc.mpg.de/groups/zweckstetter/_links/software_mars.htm
Proteome Discoverer version 2.3	ThermoScientific	https://www.thermofisher.com/uk/en/home/industrial/mass-spectrometry/liquid-chromatography-mass-spectrometry-lc-ms/lc-ms-software/multi-omics-data-analysis/proteome-discoverer-software.html; RRID: SCR_014477
NCBI PSI-BLAST	Altschul et al.^63^	https://blast.ncbi.nlm.nih.gov/Blast.cgi?PAGE_TYPE=BlastSearch&PROGRAM= blastp&BLAST_PROGRAMS=psiBlast; RRID: SCR_004870
PSIPRED	Buchan and Jones^36^	http://bioinf.cs.ucl.ac.uk/psipred/; RRID: SCR_010246
Consurf	Ashkenazy et al.^41^	https://consurf.tau.ac.il; RRID: SCR_002320
MAFFT	Katoh and Standley^64^	https://mafft.cbrc.jp/alignment/server/; RRID: SCR_011811
Jalview	Clamp et al.^65^	https://www.jalview.org; RRID: SCR_006459
Alphafold2_multimer	Evans et al., 2021b	https://github.com/deepmind/alphafold
Alphafold2_advanced	Mirdita et al.^66^	https://github.com/sokrypton/ColabFold
Bio-RAD Image lab 6.0.1	Bio-Rad	https://www.bio-rad.com/en-uk/product/image-lab-software?ID=KRE6P5E8Z; RRID: SCR_014210
GraphPad Prism	Insightful Science	https://www.graphpad.com/scientific-software/prism/; RRID: SCR_002798
ChimeraX	Petterson et al.^67^	https://www.cgl.ucsf.edu/chimerax/ RRID: SCR_015872
CASAVA	Illumina	https://support.illumina.com/sequencing/sequencing_software/bcl2fastq-conversion-software.html RRID: SCR_001802
Burrows-Wheeler Aligner	http://bio-bwa.sourceforge.net/	http://bio-bwa.sourceforge.net/ RRID: SCR_010910
Genome Analysis Toolkit	Broad Insitute	https://gatk.broadinstitute.org/hc/en-us RRID:SCR_006390
Alissa Interpret software package	Agilent	https://www.agilent.com/en/product/next-generation-sequencing/clinical-informatics-platform/alissa-interpret-930086
Other		
Econo-Pac® Chromatography Columns	BioRad	7321010
HiPrep 16/60 Sephacryl S-200 HR	Cytiva	GE17-1166-01
Superdex200 10/300 GL	Cytiva	GE17-5175-01
AKTA Protein Purification systems	Cytiva	N/A
Spex SamplePrep 6870 Freezer/Mill	Spex SamplePrep	N/A
HisTrap high performance	Cytiva	17524802
HiTrap SP high performance	Cytiva	17115201
HiTrap Heparin high performance	Cytiva	17040703
GSTrap high performance	Cytiva	GE17-5282-01
SuperSepTM Phos-TagTM	FUJIFILM Wako Pure Chemical Corporation	196-16701
Microson ultrasonic cell disrupter XL	Misonix	N/A
Branson Ultrasonics sonifier SFX250/SFX550 cell disruptors	Branson	15569624
4X Bolt LDS Sample Buffer	Invitrogen	#B0007
Glass beads diameter 0.5 mm	Sigma-Aldrich	Z250465
Bolt 4-12% Bis-Tris mini protein gel	Invitrogen	#NW04120BOX
Trans-Blot Turbo midi 0.2 μm nitrocellulose transfer packs	Bio-Rad	1704159
ChemiDoc touch imaging system	Bio-Rad	N/A
Enzymate BEH immobilized pepsin column, 2.1 × 30 mm, 5 μm	Waters	186007233
Acquity BEH C18 van-guard pre-column, 1.7 μm, 2.1 × 5 mm	Waters	186003975
Acquity UPLC BEH C18 column 1.7 μm, 100 mm × 1 mm	Waters	186002346
Acquity UPLC BEH C18 column 1.7 μm, 50 mm × 1 mm	Waters	186002344
Column with immobilized Pepsin 2.1 × 20 mm	Affipro	AP-PC-001
SYNAPT G2-Si HDMS mass spectrometer	Waters	N/A
Yeast nitrogen base without amino acids and ammonium sulfate	Sigma-Aldrich	Y1251
Bruker 700 MHz Avance II+	Bruker	N/A
Triple resonance TCI CryoProbe	Bruker	N/A
Micro SpinColumns, strong cation SP	Harvard apparatus	74-4706
Micro SpinColumns, TARGA C18	Harvard Apparatus	74-4613
CoolSafe lyophilizer	ScanVac	N/A
Ultimate U3000 HPLC	ThermoScientific	N/A
C18 Acclaim PepMap100 5 μm, 100 μm × 20 mm nanoViper	ThermoScientific	164199
C18 T3 1.8 μm, 75 μm Ç 250 mm column	Waters	186007474
Quadrupole Orbitrap mass spectrometer Q-Exactive HFX	ThermoScientific	N/A
PHERAstar FSX microplae reader	BMG Labtech	N/A
384-well plate low flange black Flat-bottom non-binding surface	Corning	3575
Illumina HiSeq 4000 platform	Illumina	N/A

### Resource Availability

#### Lead contact

Further information and requests for resources and reagents should be directed to, and will be fulfilled by Anne Bertolotti. Correspondence: aberto@mrc-lmb.cam.ac.uk.

#### Materials availability

Materials will be available from the lead contact upon request.

### Experimental Model And Study Participant Details

#### *E. coli* strains

BL21 Star (DE3) (Thermo Fisher Scientific, C601003) cells were used in this study for the production of recombinant R15B proteins.

#### Yeast strains

*S. cerevisiae* strain GP6452 cells were used in this study for the production of human eIF2 complex in Figure 1D.

#### Mammalian cells

Expi293 suspension cells were used in this study for the production of human eIF2 complex in all figures except Figure 1D. Human embryonic kidney 293T cells (HEK 293T) were used in this study for cellular assays.

### Method Details

#### Cloning, protein expression, and purification

##### Cloning, expression and purification of R15B fragments

To express R15B constructs in mammalian cells, R15B constructs (key resources table) were cloned from human cDNA using the Gibson Assembly method^69^ into the mammalian expression vector PXJ41,^70^ which contains a single amino termini FLAG tag. R15B mutants were ordered as GeneArt Strings DNA fragments (Life Technologies Ltd) with replacement of following residues with alanine: 425-430 (H1A), 449-455 (EDA), 477-481 (H2A) 565-569 (H3A). The R15B N423D mutants were generated using QuickChange II Site-Directed mutagenesis kit (Agilent, 200523) following manufacturer’s instructions.

To express recombinant R15B in *E. coli* for Figures 1C and 1D, R15B (414-613, 414-664 and 632-705) constructs were cloned into pET-47b (Merck Millipore, 71461) producing a His_6_-3C-R15B fusion protein. All constructs were expressed in BL21 Star (DE3) *E. coli* (Thermo Fisher Scientific, C601003) and purified from inclusion bodies in Luria Broth (LB) supplemented with 50 μg/ml kanamycin (Sigma-Aldrich, 60615). Cells were grown at 37 °C until OD_600_ 0.5 before induction of protein expression with 200 μM IPTG for 4 hours at 37 °C. Cells were harvested by centrifugation at 5000 RCF, 20 min and resuspended in cold lysis buffer at a 1:10 ratio (1 g pellet in 10 ml lysis buffer). The lysis buffer contained 100 mM HEPES pH 7.5, 200 mM NaCl, 0.5 mM TCEP, 40 mM imidazole, 5 mM MgCl_2_ (all chemicals from Sigma-Aldrich) and was supplemented with 1 EDTA-free protease inhibitor tablet per 50 ml lysate (Sigma-Aldrich, 11873580001) and 100 μg/ml final concentration of lysozyme (Sigma-Aldrich, L6876). Resuspended cells were lysed by sonication (Branson Sonifier) performed at 4 °C (cycle ON 2 secs, cycle OFF 5 secs for a total of 5 min per 100 ml, 35% power). Bacterial lysate was next incubated with 1250 units benzonase (Sigma-Aldrich, E1014) for 30 min on ice, under gentle stirring. Cell lysate was centrifuged for 5 min at 3000 RCF to remove non-lysed cells. The supernatant was transferred to a fresh tube followed by another centrifugation at 50000 RCF for 30 min. The supernatant was discarded and pellet was washed with 30 ml cold PBS pH 7.4 (for 1l: NaCl - 7.325 g, Na_2_HPO_4_(anhydrous) - 2.36 g, NaH_2_PO_4_.2H_2_O - 1.315 g) followed by another centrifugation for 10 min at 50000 RCF. Finally, the combined pellet was resuspended in Ni-NTA purification buffer A containing 100 mM Tris pH 8, 100 mM NaCl, 0.5 mM TCEP, 20 mM imidazole, 8 M urea and incubated for 60 min at room temperature (RT) with 4 ml of Ni-NTA resin (Qiagen, 30210) pre-equilibrated in the same buffer in gravity columns (BioRad, 7321010) under gentle agitation. Protein-bound Ni-NTA resin was then washed with buffer A (20 x column volume) and subsequently incubated with buffer B containing 100 mM HEPES pH 7.5, 100 mM NaCl, 0.5 mM TCEP, 400 mM imidazole, 8M urea for 10 min followed by elution. Eluted fractions were analysed by SDS-PAGE and protein-containing fractions were pooled and dialyzed overnight against a buffer containing of 100 mM Tris pH 8, 150 mM NaCl, 5 mM DTT, 400 mM L-arginine at 4 °C. The next day, the dialyzed protein was concentrated and filtered through a 0.45 μm syringe-filter and subjected to size exclusion chromatography (SEC) on a pre-equilibrated S200 16/600 column in buffer containing 50 mM HEPES pH 7.5, 150 mM NaCl, 0.5 mM TCEP. All SEC steps were conducted on ÄKTA systems (GE Healthcare) at 4 °C. Peak fractions were tested by SDS-PAGE and protein-containing fractions were pooled and incubated with 100 μg GST-3C protease to remove the His-tag overnight at 4 °C. Protease cleavage was assessed the next day by SDS-PAGE and the protein was subjected to another SEC as described above with the addition of a 1 ml GST-Trap (GE Healthcare) in tandem to remove any traces of the GST-tagged 3C protease. Pure, untagged protein-containing fractions were concentrated up to 250 μM. This protocol yielded an average of 3.5 mg of R15B^414-613^ protein from 1l of culture.

R15B^414-664^ construct was purified as above with the exception of the SEC, which was performed with a reduced-salt buffer consisting of 50 mM Tris pH 7.4, 100 mM NaCl, 0.5 mM TCEP. This construct that only be concentrated up to 10 μM.

R15B^632-705^ construct was purified as above with the following modifications. After Ni-NTA column purification from inclusion bodies, dialysis was performed against a buffer containing 100 mM Tris pH 8, 150 mM NaCl, 5 mM DTT, 400 mM L-arginine. Subsequently, soluble protein was dialysed against a buffer containing 50 mM HEPES pH 7.5, 150 mM NaCl, 0.5 mM TCEP and concentrated up to 10 μM.

For the rest of the study, His6-3C-R15B^414-613^ (R15B^414-613^) was purified as described above with minor changes. Briefly, R15B^414-613^ was expressed in BL21 Star (DE3) E. coli (Thermo Fisher Scientific, C601003) and purified from inclusion bodies in Luria Broth (LB) supplemented with 50 μg/ml kanamycin (Sigma-Aldrich, 60615). Cells were grown at 37 °C until OD600 0.4-0.6 before induction of protein expression with 200 μM IPTG O/N at 20 °C. Cells were harvested by centrifugation at 5000 rcf, 20 min and resuspended in cold lysis buffer (100 mM HEPES pH 7.5, 150 mM NaCl, 0.5 mM TCEP, 20 mM imidazole, 5 mM MgCl_2_ (all chemicals from Sigma-Aldrich) and was supplemented with 1 EDTA-free protease inhibitor tablet per 50 ml lysate (Sigma-Aldrich, 11873580001), 2 μl benzonase nuclease and 0.2 μg/ml final concentration of lysozyme (Sigma-Aldrich, L6876). Resuspended cells were lysed by sonication performed at 4 °C, 40% intensity, 2 min (2 sec on, 8 sec off). Cell lysate was centrifuged for 20 min at 50000 rcf. The pellet was resuspended in buffer A (100 mM HEPES pH 7.5, 100 mM NaCl, 0.5 mM TCEP, 20 mM imidazole, 8 M urea) and incubated for 60 min at RT with Ni-NTA resin (Qiagen, 30210) pre-equilibrated in the same buffer. Protein-bound Ni-NTA resin was then washed with buffer A and protein was eluted with buffer B (100 mM HEPES pH 7.5, 100 mM NaCl, 0.5 mM TCEP, 400 mM imidazole, 8M urea). Eluted fractions were analysed by SDS-PAGE and protein-containing fractions were pooled and dialysed overnight against a buffer containing of 50 mM HEPES pH 7.5, 150 mM NaCl, 0.5 mM TCEP, 400 mM L-arginine at 4 °C. Protein was concentrated, and subjected to size exclusion chromatography (SEC) on a S200 16/600 column (Cytiva, GE17-1166-01) in buffer containing 50 mM HEPES pH 7.5, 150 mM NaCl, 0.5 mM TCEP. Peak fractions were tested by SDS-PAGE.

##### His-tag cleavage

To cleave His-tag from His6-3C-R15B^414-613^, 100 μg of GST-3C protease was added to the protein followed by O/N incubation at 4 °C. Protease cleavage was assessed by SDS-PAGE and the protein was subjected to another SEC the addition of a 1 ml GST-Trap (Cytiva, GE17-5175-01) in tandem to remove any traces of the GST-tagged 3C protease (Cytiva, GE27-0843-01). His6-3C-R15B 414-613 construct

All R15B constructs and their respective expression vectors are listed in the STAR table.

##### Purification of human eIF2 from yeast

For Figure 1D, the human eIF2 complex was purified from *S. cerevisiae* strain GP6452 as established and described in ^32,71^. Briefly, yeast cells were grown to saturation in synthetic complete media lacking leucine, tryptophan and uracil and supplemented with 2% glucose. A 20-liter yeast culture was grown in yeast extract peptone (YEP) media supplemented with 2% galactose and 0.2% glucose for 24 hours at 30 °C. Cells were harvested by centrifugation at 5000 RCF, 20 min and resuspended in lysis buffer (100 mM Tris, pH 8.5, 300 mM KCl, 5 mM MgCl_2_, 0.1% NP-40, 5 mM imidazole, 10% glycerol, 2 mM DTT, 1 EDTA-free protease inhibitor tablet per 50 ml lysate (Sigma-Aldrich, 11873580001), 1 μg/ml each aprotinin, leupeptin, pepstatin A (all from Sigma-Aldrich: 10236624001, L2884, P5318 respectively). Resuspended cells were frozen as droplets in liquid nitrogen and subsequently lysed using a freezer mill cryogenic grinder (Spex SamplePrep). The freezer mill allowed grinding of the yeast cells into a fine powder at cryogenic temperatures preserving protein structure and protein assemblies. The lysate was centrifuged twice at 50,000 RCF for 30 min at 4°C. The eIF2 complex was purified by tandem affinity purification utilising His_6_-eIF2β and FLAG-eIF2α, as described below. Lysate was applied to a 5 ml HisTrap HP column (GE Healthcare) equilibrated in buffer (100 mM HEPES, pH 7.5, 100 mM KCl, 5 mM MgCl_2_, 0.1% NP-40, 5% glycerol, 1 mM DTT, 13 protease inhibitor cocktail (Sigma-Aldrich, 11873580001), 1 μg/ml each aprotinin, leupeptin, pepstatin A), followed by a 20 x column-volumes wash. eIF2 complex was eluted using a 50 ml linear gradient of 5 mM to 500 mM imidazole. Protein containing fractions were combined and incubated with anti-FLAG M2 magnetic affinity beads (Sigma-Aldrich, M8823), followed by washes with FLAG wash buffer (100 mM HEPES, pH 7.5, 100 mM KCl, 5 mM MgCl_2_, 0.1% NP-40, 5% glycerol, 1 mM TCEP, 1 x protease inhibitor cocktail, 1 mg/ml each aprotinin, leupeptin, pepstatin A) and eluted with FLAG elution buffer containing 100 μg/ml 3 x FLAG peptide (Sigma-Aldrich, F4799). The eIF2 complex was further purified by SEC using an S200 10/300 column pre-equilibrated in buffer containing 50 mM HEPES pH 7.5, 100 mM KCl, 5 mM MgCl_2_, 0.5 mM TCEP, 1% glycerol. Peak fractions were tested by SDS-PAGE and appropriate protein-containing fractions were pooled and concentrated up to 200 μM.

##### Purification of human eIF2 from Expi293 cells

The purification of eIF2 was performed similarly as described in Kashiwagi et al.^72^ Briefly, the α-, β-, and γ-subunits of human eIF2, and the eIF2-specific chaperone protein human Cdc123 were co-expressed in Expi293 suspension cells, using the four pEBMulti-Neo plasmid vectors (Wako), and eIF2γ was expressed with C-terminal FLAG and His8 tags. The cells were lysed for 30 mins on ice in buffer A (20 mM MES-KOH buffer (pH 6.0), containing 150 mM KCl, 1 mM MgCl2, 10% (v/v) glycerol, and 1 mM TCEP) supplemented with 20 mM imidazole, 0.5 mM EDTA, 0.1%(v/v) Triton X-100, and protease inhibitors. The cell lysate was centrifuged and the supernatant was applied to a HisTrap (Cytiva) column equilibrated with buffer A supplemented with 20 mM imidazole and 0.5 mM TCEP, and eluted with a linear gradient of 20–500 mM imidazole. The fraction containing eIF2 was collected and applied to a HiTrap SP (Cytiva) column equilibrated with buffer A, and eluted with a linear gradient of 200–640 mM KCl. After three-fold dilution with buffer B (20 mM HEPES-KOH buffer (pH 7.5) containing 100 mM KCl, 0.1 mM MgCl2, 10% (v/v) glycerol, and 0.5 mM TCEP), the sample was applied to a HiTrap Heparin (Cytiva) column equilibrated with buffer B, and eluted with a linear gradient of 0.2–1 M KCl. The fraction containing eIF2 was further purified on a Superdex200 (GE Healthcare) column equilibrated with buffer (50 mM HEPES pH 7.5, 100 mM KCl, 5 mM MgCl2, 0.5 mM TCEP, 1% Glycerol). Peak fractions were analysed on SDS-PAGE.

#### Mammalian cell culture and immunoprecipitations

##### Cell culture

Human embryonic kidney 293T cells (HEK 293T) were grown in a humidified incubator with 5% CO_2_ at 37°C, cultured in Dulbecco’s Modified Eagle’s Media (DMEM, Sigma, D5796) supplemented with 10% fetal bovine serum (FBS, Gibco 10270), 2 mM L-glutamine (Gibco, 25030), 100 U/mL penicillin and 100 μg/mL streptomycin (Gibco, 15140122).

##### Transient PEI transfection

HEK 293T cells were seeded at 0.8x10^6^ cells per 10 cm^2^ dish and incubated for 24 hours. 4 μg of the indicated R15B constructs, in the PXJ41 vector, were mixed with 800 μl of Opti-MEM media (Gibco, 11058) and 12 μl PEI transfection reagent (Polysciences, 24765) and incubated for 20 min at RT. The transfection mixture was added drop wise onto the cells and mixed gently. Cells were incubated for a further 24 hours.

##### Cell lysates

Cells were washed with 5 ml ice-cold PBS prior to centrifugation for 5 min at 300 RCF, 4 °C. 800 μl of lysis buffer (50 mM Tris-HCl pH 7.4, 10 mM NaCl, 100 mM KCl 0.1 μM CaCl_2_, 0.5 mM MgCl_2_, 0.5 mM TCEP, EDTA-free complete protease tablet (Roche, 04639159001) was used to lyse the cell pellet. Lysates were sonicated for 3x 3 secs using a Microson ultrasonic cell disrupter XL (Misonix) on ice. Lysates were subjected to microcentrifugation at 4 °C for 12 min at 16000 RCF and the supernatants were transferred to fresh tubes.

##### FLAG immunoprecipitation

Per condition, 10 μl of anti-FLAG M2 magnetic beads (Sigma-Aldrich, M8823-1ML) were pre-equilibrated in lysis buffer (see above). The beads were dispatched into 700 μl of lysates and incubated overnight at 4 °C on a rotating wheel. Samples were washed three times with lysis buffer before elution of the proteins with 50 μl of 1X BOLT LDS (Novex #B0007) containing 100 mM DTT, boiled at 95 °C for 10 min. 10 μl of both lysates (input) and immunoprecipitated samples (FLAG-IP) were analysed by immunoblots.

##### Dephosphorylation activity assays

HEK293T cells were seeded at 1x10^6^ per 10cm^2^ dish. The day after seeding, cells were transfected with 1 μg of each R15 full-length construct (WT, H1A and N432D) for 24 hours and lysed as described above. Constructs were transfected at 1 μg each to provide differentiation between construct activities in this overexpression system. For Figure 5D, p-eIF2α was assessed on a different membrane to total eIF2α with Ponceau S solution (Sigma, P7170) utilised to assess transfer quality and equal loading between membranes. For Figure 6F, detection of total eIF2α was carried out on the immunoblot used for detecting p-eIF2α following incubation in stripping buffer (ThermoFisher Scientific, 2105). Bands were quantified using Image Lab Software (Bio-Rad) and the statistical analysis was performed using GraphPad Prism 8 using one-way ANOVA.

##### FLAG-immunoprecipitation of yeast lysates and binding studies

Frozen yeast cells (GP6452 strain) (5 g) were resuspended in PBS (10 ml) and mixed with 2 g of fine glass beads (Sigma-Aldrich, Z250465). Yeast cells were lysed by mechanical glass bead disruption (vortexing for 30 sec and resting for 30 sec on ice) for 10 min in total. The mixture was then clarified by centrifugation at 55000 RCF for 15 min at 4 °C. Clarified lysate was equally divided and incubated with 50 μl of pre-equilibrated FLAG M2 magnetic affinity beads (Sigma-Aldrich, M8823) for 2 hours at 4 °C. Samples were washed four times with PBS for 10 min. Then, 2.5 μM of R15B protein was added in a total volume of 1 ml of PBS with 1 mg/ml of BSA. All reactions were incubated at 4 °C for 1 hour with gentle rotation. Subsequently, beads were washed four times with PBS for 10 min. Samples were incubated for 30 min at 4 °C and eluted from the beads using 50 μl of PBS with 100 μg/ml 3x FLAG elution peptide (Sigma-Aldrich, F4799). Samples were then mixed with sample loading buffer (Novex #B0007) containing 100 mM DTT (Sigma-Aldrich, 10708984001) and were loaded on an SDS-PAGE followed by immunoblotting with appropriate antibodies.

##### Analytical size exclusion chromatography

R15B^414-613^ or eIF2 were subjected to SEC on a pre-equilibrated S200 10/300 column (GE Healthcare) with buffer containing 50 mM HEPES pH 7.5, 100 mM KCl, 5 mM MgCl_2_, 0.5 mM TCEP, 1% Glycerol using an Akta Pure (GE Healthcare). For the complex formation, R15B^414-613^ and eIF2 complex were mixed together and incubated on ice for 30 min. The sample was injected to SEC on Superose 6 3.6/300 column (Cytiva). Fractions were then analysed by SDS-PAGE.

##### Fluorescence anisotropy

The reactions were completed in a buffer of 50 mM HEPES pH 7.5, 100 mM KCl, 0.5 mM TCEP, 0.05% (v/v) Tween-20. To analyse the binding of R15B to eIF2 complex, a sixteen of twofold dilution series of eIF2 from 250 nM were prepared and mixed 1:1 with fluorescently labelled R15B^414-613^ (NT-495; Nanotemper MO-L003) at 20 nM. Measurements were performed with a PheraStar plate reader FSX plate reader (BMG Labtech) using an optic module for λ_ex_ = 485 nm, λ_em_ = 520 nm. Reactions were carried out in a total volume of 40 μl at 25 °C in a black, flat-bottom, non-binding surface 384-well plate (Corning). The experiment was performed as technical triplicates and data were analysed in PRISM 9.5.1 (GraphPad Software).

##### Immunoblots and antibodies

Proteins were resolved on Bolt 4-12% Bis-Tris Plus gel (Invitrogen, #NW04120BOX) in 1X MES running buffer at 120 V for 70 min with 2 μL of Protein Precision Plus Dual Colour Standards (#161-0374) loaded on each gel. Proteins were transferred onto a nitrocellulose membrane (Bio-Rad, 1704159) using a Trans-Blot Turbo System (BioRad). Ponceau S solution (Sigma, P7170) was used to assess transfer quality and equal loading. Membranes were blocked for 1 hour at RT in TBS with 0.025% Tween 20 (Sigma, P1379) (TBS-T) and 5% milk. Membranes were rinsed 3 times with TBS-T and incubated with the relevant primary antibody diluted in 5% BSA in TBS-T overnight at 4 °C. Following three washes with TBS-T, membranes were incubated with the appropriate horseradish peroxidase-conjugated secondary antibody (Promega, W4011, W4021) diluted in TBS-T with 5% milk for 1 hour at RT. Membranes were washed three times with TBS-T and once with TBS before imaging. Amersham ECL Prime detection reagent kit (GE Healthcare Life Sciences, RPN2232) was used to detect chemiluminescence with ChemiDoc Touch imagingsiystem (Bio-Rad). The following primary antibodies were used: PP1 (Santa Cruz, Sc-7482, 1:1000), total eIF2α (Abcam, Ab26197, 1:1000), P-eIF2α (Abcam, Ab32157, 1:1000), Vinculin (Cell Signaling Technology, 4650S, 1:5000), FLAG (Sigma-Aldrich, F7425, 1:1000), R15B (In house 3E11, 1:1000), R15B (Proteintech, 4634-1-AP, 1:2000 dilution).

#### Hydrogen-deuterium exchange mass spectrometry of eIF2 and eIF2 with R15B

##### Sample preparation and data acquisition

R15B^414-613^ and eIF2 complex were prepared separately as described above. Both samples were dialyzed separately against buffer containing 50 mM HEPES pH 7.4, 100 mM KCl, 5 mM MgCl_2_, 0.5 mM TCEP overnight at 4 °C. HDX-MS experiments were performed with final equimolar concentration of 5.5 μM for both R15B^414-613^ and eIF2. An aliquot of 5 μl was incubated with 45 μl of D_2_O buffer atRT for 3, 30, 300 and 3000 secs in triplicate. The labelling reaction was quenched by adding chilled 2.4% v/v formic acid in 2 M guanidinium hydrochloride and immediately frozen in liquid nitrogen. Samples were stored at -80 °C prior to analysis.

The quenched protein samples were rapidly thawed and subjected to proteolytic cleavage by pepsin followed by reversed phase HPLC separation. Briefly, the protein was passed through an Enzymate BEH immobilized pepsin column, 2.1 x 30 mm, 5 μm (Waters, UK) at 200 μl/min for 2 min and the peptic peptides trapped and desalted on a 2.1 x 5 mm C18 trap column (Acquity BEH C18 Vanguard pre-column, 1.7 μm, Waters, UK). Trapped peptides were subsequently eluted over 12 min using a 5-36% gradient of acetonitrile in 0.1% v/v formic acid at 40 μl/min. Peptides were separated on a reverse phase column (Acquity UPLC BEH C18 column 1.7 μm, 100 mm x 1 mm (Waters, UK). Peptides were detected on a SYNAPT G2-Si HDMS mass spectrometer (Waters, UK) acquiring over a *m/z* of 300 to 2000, with the standard electrospray ionization (ESI) source and lock mass calibration using [Glu1]-fibrino peptide B (50 fmol/μl). For protein identification, mass spectra were acquired in MSE mode. The mass spectrometer was operated at a source temperature of 80°C and a spray voltage of 2.6 kV. Spectra were collected in positive ion mode. The other spectrometer parameters were as follows: cone voltage 30 V, desolvation gas 650 (L/h), collision energy ramp 20-40 V. IMS was used for R15B and R15B with eIF2. HDX experiments were done in triplicate.

##### Data analysis

Peptide identification was performed by MS^e^ ^73^ using an identical gradient of increasing acetonitrile in 0.1% v/v formic acid over 12 min. The resulting MS^e^ data were analysed using Protein Lynx Global Server software (Waters, UK) with an MS tolerance of 5 ppm.

Mass analysis of the peptide centroids was performed using DynamX software (Waters, UK) with the following thresholds: minimum intensity 2000, minimum products per amino acid 0.3, minimum consecutive products 1, maximum ppm 5. Only peptides with a score >6.4 were considered. The first round of analysis and identification was performed automatically by the DynamX software, however, all peptides (deuterated and non-deuterated) were manually verified at every time point for the correct charge state, presence of overlapping peptides, and correct retention time. Deuterium incorporation was not corrected for back-exchange and represents relative, rather than absolute changes in deuterium levels. Changes in H/D amide exchange in any peptide may be due to a single amide or a number of amides within that peptide. All time points in this study were prepared at the same time and individual time points were acquired on the mass spectrometer on the same day. The values reflecting the experimental mass of each peptide in all possible states, replicates, time points, and charge states were exported from DynamX 3.0, and further data analysis and plots were carried out using HaDeX^74^ Briefly, Wood’s plots are plotted with the fraction exchanged difference between two states. Confidence intervals calculated with two-tailed t-test at 0.98 and 0.99 as error bars representing the combined uncertainty for each data point are shown. Detailed calculations are described in the documentation: https://hadexversum.github.io/HaDeX/articles/datafiles.html. We highlighted with blue (increased protection) and red (decreased protection) coloured rectangles the peptide with the Δ fraction exchange greater than 5% present in at least two different time points or the region with at least three peptides with the Δ fraction exchange greater than 5% in one time point.

#### HDX-MS of R15B and R15B with eIF2

##### Buffer and pH

For physiological pH buffer of 50 mM HEPES pH 7.5, 100 mM KCl, 5 mM MgCl2 and 0.5 mM TCEP was used. For low pH buffer (pD 6.0) of 20 mM MES-KOH, 100 mM KCl, 5 mM MgCl2 and 0.5 mM TCEP was used. pH of the buffers was measured with a Mettler-Toledo pH meter.

##### Sample preparation

Peptide list was obtained by diluting 5 μl of R15B^414-613^ tenfold into a physiological pH, non-deuterated buffer. The sample was acidified by mixing with 10 μl chilled quenching buffer (2 M glycine pH 2.5, 2 M guanidinium hydrochloride and 10 mM TCEP) and subjected to proteolytic cleavage by pepsin followed by reversed phase HPLC separation and mass spectrometry detection. Briefly, the protein was passed through a 2.1 x 20 mm immobilized pepsin column (Affipro) at 100 μl/min for 2 min and the peptic peptides trapped and desalted on a 2.1 x 5 mm C18 trap column (Acquity BEH C18 Van-guard pre-column, 1.7 μm, (Waters)). Peptides were separated on a reverse phase column (Acquity UPLC BEH C18 column 1.7 μm, 50 mm x 1 mm, (Waters)) with a 6-35% gradient of acetonitrile in 0.1% v/v formic acid at 90 μl/min. Total time of a single run was 12 min. All capillaries, valves, and columns were maintained at 0.1°C inside an HDX cooling chamber, while the pepsin column was kept at 13°C inside the temperature-controlled digestion compartment. Peptides were detected on a SYNAPT G2-Si HDMS mass spectrometer (Waters) acquiring over a m/z of 50 to 2000, with the standard electrospray ionization (ESI) source and lock mass calibration using [Glu1]-fibrino peptide B (50 fmol/μl). The mass spectrometer was operated at a source temperature of 80°C and a spray voltage of 2.6 kV. Spectra were collected in positive ion mode. Peptides were identified using ProteinLynx Global Server (PLGS) software (Waters). The list of identified peptides containing peptide m/z, charge, and retention time was further processed with the DynamX v. 3.0 program (Waters). For HDX experiment, protein sample (R15B^414-613^ or R15B^414-613^ + eIF2 complex) was diluted in the reaction buffer containing deuterium oxide (D_2_O, VWR Chemicals). Five microliters of protein stock solution were mixed with 45 μl D_2_O reaction buffer, and incubated for 3, 30, 60 and 300 sec. The labelling reaction was quenched by adding 10 μL chilled quenching buffer (2 M glycine pH 2.5, 2 M guanidinium hydrochlo-ride and 10 mM TCEP) and immediately frozen in liquid nitrogen. Samples were stored at -80 °C prior to analysis. The quenched protein samples were rapidly thawed and manually injected into the ACQUITY UPLC system (Waters). Further digestion, HPLC, and MS analysis were carried out as described for the non-deuterated sample. For ‘maximally labelled’ control sample, the 5 μl of protein stock was mixed with 45 μl of D_2_O reaction buffer, incubated for 24 hours at RT, mixed with quenching buffer, and analysed as described above. HDX experiments were done at least in triplicate.

##### Data analysis

A peptide list was created for R15B^414-613^ using the DynamX 3.0 software based on PLGS peptide identifications, with the following acceptance criteria: minimum intensity threshold 3000, minimum fragmentation products per amino acids for precursor 0.3, maximum mass difference between measured and theoretical value for parent ions 5 ppm. Centroids of the mass envelopes were obtained. The first round of analysis and identification was performed automatically by the DynamX software, however, all peptides were manually verified at every time point for the correct charge state, presence of overlapping peptides, and correct retention time. The values reflecting the experimental mass of each peptide in all possible states, replicates, time points, and charge states were exported from the DynamX 3.0, and further data analysis and plots were carried out using HaDeX^74^ as described above. We highlighted with blue the statistically significant regions with greatest differences.

##### Data and Software availability

The full HDX-MS datasets are included as Supplementary datasets.

#### NMR

##### Isotopic labelling for NMR

Isotopically labelled R15B^414-613^ was overexpressed in M9 media (6 g/l Na_2_HPO_4_, 3 g/l KH_2_PO_4_, 0.5 g/l NaCl) supplemented with 1.7 g/l yeast nitrogen base without NH_4_Cl and amino acids (Sigma-Aldrich, Y1251). 1 g/l ^15^NH_4_Cl and 4 g/l ^13^C-glucose were supplemented for ^15^N and ^13^C labelling respectively. Additionally, filtered-sterile solutions of 2 mM MgSO_4_ (final concentration in M9 media), 25 mg FeSO_4_.7H_2_O, and 1 ml vitamin mix (100 ml vitamin mix stock solution contains riboflavin 100 mg (Sigma-Aldrich, R9504), niacinamide 100 mg (Sigma-Aldrich, N5535), pyridoxine hydrochloride 10 mg (Sigma-Aldrich, P9755), thiamine 100 mg (Sigma-Aldrich, T1270) were added to the media. Cells were grown in M9 media as described for LB media above with the following exception: cells were grown at 37 °C until OD_600_ 0.6 before induction of protein expression with 200 μM IPTG for 18 hours at 22 °C. Isotopically labelled protein was purified as described above for the native protein.

##### NMR spectroscopy

Solution-state NMR data were acquired at 281.5K on a Bruker Avance II+ spectrometer operating at a proton resonance frequency of 700MHz and fitted with a 5mm TCI triple resonance cryoprobe. All NMR samples were prepared in 50 mM HEPES at pH 7.5, with 150 mM NaCl, 10mM DTT, and with 5% D_2_O as lock solvent. The concentration of R15B was set to 150 μm, to probe interaction hotspots, an additional 10% of unlabelled eIF2 (15μM) was added under identical conditions.

*Backbone assignment*. Assignment of backbone H_N_, N, Cα, Cβ and C’ resonances of isotopically enriched ^15^N,^13^C R15B^414-613^ were obtained with the following triple resonance experiments: HNCO, HN(CA)CO, HNCACB, CBCA(CO)NH, HN(CAN)NH and HN(COCA)NNH (Bruker pulse sequence library). Data collection with 15-25 % non-uniform sampling yielded high-resolution 3D datasets which were processed either in Topspin versions 3.2 or 4 (Bruker) or using NMRPipe^75^ in combination with the MddNMR package^60^ for compressed sensing reconstruction. Data analysis employed Sparky 3,^61^ POKY^76^ and MARS.^62^

*Secondary chemical shifts*. Secondary chemical shift analysis was performed to probe secondary structure elements. Random coil Ca values for R15B^414-613^ were calculated according to^77–79^ and subtracted from the experimentally derived values to arrive at ΔCα values. The observed secondary shifts are negative for residues in an extended backbone conformation, positive values indicate a preference for helical secondary structure.

^*15*^*N backbone dynamics measurements*. NMR relaxation measurements included the acquisition of ^15^N longitudinal T_1_ (1/R_1_), ^15^N transverse T_2_ (1/R_2_), ^15^N {^1^H} NOE and ^15^N h_xy_ (^1^H-^15^N dipolar/^15^N chemical shift anisotropy (CSA) cross-correlated relaxation rate) measurements. T_1_,T_2_ relaxation experiments were acquired using standard INEPT based pseudo-3D pulse sequences (Bruker) in an interleaved manner with a 5 sec recovery delay preceded by a temperature compensation module.

^15^N T_1_ experiments were collected with 12 relaxation delays (0.01 (*2), 0.02, 0.04, 0.08, 0.12, 0.16, 0.32, 0.64, 1.28 secs), ^15^N T_2_ experiments with CPMG interpulse delays of 450 ms (spinlock field n_cpmg_ = 1.1 kHz) included 12 relaxation delays (8.48 repeated twice, 16.96, 33.92, 50.88, 67.84, 101.76, 135.68, 169.6, 203.52, 237.44, 271.36 msecs). Relaxation data were analysed in POKY^76^ based on peak intensities. Heteronuclear Overhauser enhancements were measured after 5 secs proton irradiation and {^1^H}-^15^N NOE values were calculated as the peak intensity difference I/I_o_ between saturated and reference spectra and repeated twice for error estimation. The ^15^N η_xy_ cross-correlated relaxation rates were measured with relaxation delays (2Δ) of 60 and 100ms according to^80^ and fitted to the following equation:

$${\text{I}}_{\text{A}}/{\text{I}}_{\text{B}}=\tanh\left(2\text{Δ}\eta_{\text{xy}}\right)$$

Where I_A_, I_B_ are the signal intensities obtained with pulse schemes A and B, which separately address relaxation of ^15^N downfield and upfield doublet components. The exchange-free R_2_ relaxation rate R_2_^0^ was calculated according to^40^:

$${{\text{R}}_{2}}^{0}=\kappa \eta_{\text{xy}}+1.3\sigma$$

With

$$\sigma =(\text{NOE}-1)\times {\text{R}}_{1}\times \gamma \text{N}/\gamma \text{H}$$

γN and γH are the gyromagnetic ratios of ^15^N and ^1^H, the value κ was set empirically to a value of 1.45. Chemical exchange contributions R_ex_ were calculated as

$${\text{R}}_{\text{ex}}={\text{R}}_{2,\text{cpmg}}-{{\text{R}}_{2}}^{0}$$

#### Whole-exome sequencing

Trio exome capturing was carried out using Agilent SureSelect Target Enrichment Clinical Research Exome V2 (Agilent Technologies, Santa Clara, CA, USA). Sequencing (paired-end 150bp) was performed by the Illumina HiSeq 4000 platform (Illumina, San Diego, CA, USA). Data was demultiplexed by Illumina Software CASAVA. Reads are mapped to the genome (build hg19/GRCh37) with the program BWA (reference: http://bio-bwa.sourceforge.net/). Variants are detected with the Genome Analysis Toolkit (reference: http://www.broadinstitute.org/gatk/). Subsequently, variants were filtered with the Alissa Interpret software package (Agilent technologies) on quality (read depth ≥ 10), frequency in databases (≥1% in 200 alleles in dbSNP, ESP6500, the 1000 Genome project or the ExAC database) and location (within an exon or first/last 10 bp of introns). Variants were further selected based on three inheritance models (de novo autosomal dominant, autosomal recessive and X-linked recessive). With this strategy, we identified rare variants in *PPP1R15B, NVL, SHROOM2* and *CCDC110* genes. Based on the clinical phenotype the homozygous variants in *PPP1R15B* was prioritized and confirmed by Sanger sequencing.

#### Bioinformatics

NCBI-NR was searched using PSI-BLAST^63^ to identify sequence homologs of R15B. Selected 150 sequences were aligned, after which the alignment was manually corrected and used as an input for conservation scores calculation. The conservation scores were computed using Consurf^41^ and mapped on the sequence of human R15B. Multiple sequence alignments of R15 repeats were produced with MAFFT,^64^ manually corrected and visualized using Jalview.^65^ The model of R15B^414-500^, eIF2α 1-315, eIF2β 167-333, eIF2γ 1-472 was generated using AlphaFold2 version 2.3.1 multimer mode, using amber relaxation and no template.^37,38,66^ An AlphaFold2 model of NTD-eIF2α, PP1 and R15B^637-713^ was generated with a template (PDB:7nzm) and subsequent relaxation applied. The models were superimposed on the NTD of eIF2α for a composite model. Figures 7 and S6 were created with ChimeraX.^67^

### Quantification And Statistical Analysis

Western blots were quantified using Image Lab Software (Bio-Rad) and the statistical analysis was performed using GraphPad Prism 8 using one-way ANOVA. Statistical analysis for HDX-MS experiments is described in the method details section.

## Supplementary Material

Supplementary material

Table S1

## Data Availability

NMR datasets have been deposited in BMRB and HDX-MS data have been deposited to the ProteomeXchange Consortium via the PRIDE partner repository^68^ with the dataset identifier PXD047538. Accession numbers are listed in the key resources table. Uncropped, original western blot images have been deposited at Mendeley and are publicly available as of the date of publication. The DOI (https://doi.org/10.17632/v26bzg9833.1) is also listed in the key resources data. This paper does not report original code. Any additional information required to reanalyse the data reported in This paper is available from the lead contact upon request.

## Highlights

- R15B binds the far end of the 125 kDa eIF2 substrate relative to the phosphosite
- R15B is largely disordered and binds eIF2 through three short helical elements
- A homozygote variant impaired in substrate binding causes microcephaly

## In brief

Fatalska et al. revealed how the intrinsically disordered PP1 non-catalytic subunit PPP1R15B (R15B) captures its full trimeric eIF2 substrate by binding the far end of the complex relative to the phosphosite using three short helical elements. A homozygote variant in R15B impaired in substrate binding causes microcephaly.

## References

[1] Cohen P (2002). The origins of protein phosphorylation. Nat Cell Biol.

[2] Humphrey SJ, James DE, Mann M (2015). Protein phosphorylation: A major switch mechanism for metabolic regulation. Trends Endocrinol Metab.

[3] Chen MJ, Dixon JE, Manning G (2017). Genomics and evolution of protein phosphatases. Sci Signal.

[4] Shi Y (2009). Serine/threonine phosphatases: mechanism through structure. Cell.

[5] Brautigan DL (2013). Protein Ser/Thr phosphatases - the ugly ducklings of cell signalling. FEBS Journal.

[6] Goldberg J, Huang HB, Kwon YG, Greengard P, Nairn AC, Kuriyan J (1995). Three-dimensional structure of the catalytic subunit of protein serine/threonine phosphatase-1. Nature.

[7] Egloff MP, Cohen PT, Reinemer P, Barford D (1995). Crystal structure of the catalytic subunit of human protein phosphatase 1 and its complex with tungstate. J Mol Biol.

[8] Heroes E, Lesage B, Görnemann J, Beullens M, Van Meervelt LV, Bollen M (2013). The PP1 binding code: a molecular-lego strategy that governs specificity. FEBS Journal.

[9] Virshup DM, Shenolikar S (2009). From promiscuity to precision: protein phosphatases get a makeover. Mol Cell.

[10] Roy J, Cyert MS (2009). Cracking the phosphatase code: docking interactions determine substrate specificity. Sci Signal.

[11] Egloff MP, Johnson DF, Moorhead G, Cohen PT, Cohen P, Barford D (1997). Structural basis for the recognition of regulatory subunits by the catalytic subunit of protein phosphatase 1. EMBO J.

[12] Terrak M, Kerff F, Langsetmo K, Tao T, Dominguez R (2004). Structural basis of protein phosphatase 1 regulation. Nature.

[13] Hurley TD, Yang J, Zhang L, Goodwin KD, Zou Q, Cortese M, Dunker AK, DePaoli-Roach AA (2007). Structural basis for regulation of protein phosphatase 1 by inhibitor-2. J Biol Chem.

[14] Zhou Y, Millott R, Kim HJ, Peng S, Edwards RA, Skene-Arnold T, Hammel M, Lees-Miller SP, Tainer JA, Holmes CFB (2019). Flexible tethering of ASPP proteins facilitates PP-1c catalysis. Structure.

[15] O’Connell N, Nichols SR, Heroes E, Beullens M, Bollen M, Peti W, Page R (2012). The molecular basis for substrate specificity of the nuclear NIPP1:PP1 holoenzyme. Structure.

[16] Choy MS, Hieke M, Kumar GS, Lewis GR, Gonzalez-DeWhitt KR, Kessler RP, Stein BJ, Hessenberger M, Nairn AC, Peti W (2014). Understanding the antagonism of retinoblastoma protein dephosphorylation by PNUTS provides insights into the PP1 regulatory code. Proc Natl Acad Sci USA.

[17] Choy MS, Yusoff P, Lee IC, Newton JC, Goh CW, Page R, Shenolikar S, Peti W (2015). Structural and functional analysis of the GADD34: PP1 eIF2α phosphatase. Cell Rep.

[18] Hendrickx A, Beullens M, Ceulemans H, Den Abt T, Van Eynde A, Nicolaescu E, Lesage B, Bollen M (2009). Docking motif-guided mapping of the interactome of protein phosphatase-1. Chem Biol.

[19] Bertolotti A (2018). The split protein phosphatase system. Biochem J.

[20] Cohen PT (2002). Protein phosphatase 1 targeted in many directions. J Cell Sci.

[21] Ruff KM, Pappu RV (2021). AlphaFold and implications for intrinsically disordered proteins. J Mol Biol.

[22] Babu MM, van der Lee R, de Groot NS, Gsponer J (2011). Intrinsically disordered proteins: regulation and disease. Curr Opin Struct Biol.

[23] Fedoryshchak RO, Přechová M, Butler AM, Lee R, O’Reilly N, Flynn HR, Snijders AP, Eder N, Ultanir S, Mouilleron S (2020). Molecular basis for substrate specificity of the Phactr1/PP1 phosphatase holoenzyme. eLife.

[24] Ragusa MJ, Dancheck B, Critton DA, Nairn AC, Page R, Peti W (2010). Spinophilin directs protein phosphatase 1 specificity by blocking substrate binding sites. Nat Struct Mol Biol.

[25] Sonenberg N, Hinnebusch AG (2009). Regulation of translation initiation in eukaryotes: mechanisms and biological targets. Cell.

[26] Wek RC (2018). Role of eIF2α kinases in translational control and adaptation to cellular stress. Cold Spring Harb Perspect Biol.

[27] Chen R, Rato C, Yan Y, Crespillo-Casado A, Clarke HJ, Harding HP, Marciniak SJ, Read RJ, Ron D (2015). G-actin provides substrate-specificity to eukaryotic initiation factor 2a holophosphatases. eLife.

[28] Yan Y, Harding HP, Ron D (2021). Higher-order phosphatase–substrate contacts terminate the integrated stress response. Nat Struct Mol Biol.

[29] Hodgson G, Andreeva A, Bertolotti A (2021). Substrate recognition determinants of human eIF2α phosphatases. Open Biol.

[30] Carrara M, Sigurdardottir A, Bertolotti A (2017). Decoding the selectivity of eIF2α holophosphatases and PPP1R15A inhibitors. Nat Struct Mol Biol.

[31] Dar AC, Dever TE, Sicheri F (2005). Higher-order substrate recognition of eIF2α by the RNA-dependent protein kinase PKR. Cell.

[32] de Almeida RA, Fogli A, Gaillard M, Scheper GC, Boesflug-Tanguy O, Pavitt GD (2013). A yeast purification system for human translation initiation factors eIF2 and eIF2Bε and their use in the diagnosis of CACH/VWM disease. PLoS One.

[33] Britt HM, Cragnolini T, Thalassinos K (2022). Integration of mass spectrometry data for structural biology. Chem Rev.

[34] Goswami D, Devarakonda S, Chalmers MJ, Pascal BD, Spiegelman BM, Griffin PR (2013). Time window expansion for HDX analysis of an intrinsically disordered protein. J Am Soc Mass Spectrom.

[35] Dyson HJ, Wright PE (2021). NMR illuminates intrinsic disorder. Curr Opin Struct Biol.

[36] Buchan DWA, Jones DT (2019). The PSIPRED Protein Analysis Workbench: 20 years on. Nucleic Acids Res.

[37] Evans R, O’Neill M, Pritzel A, Antropova N, Senior A, Green T, Žídek A, Bates R, Blackwell S, Yim J, Ronneberger O (2021). Protein Complex prediction with AlphaFold-Multimer. biorxiv.

[38] Jumper J, Evans R, Pritzel A, Green T, Figurnov M, Ronneberger O, Tunyasuvunakool K, Bates R, Žídek A, Potapenko A (2021). Highly accurate protein structure prediction with AlphaFold. Nature.

[39] Mirdita M, Schütze K, Moriwaki Y, Heo L, Ovchinnikov S, Steinegger M (2022). ColabFold: making protein folding accessible to all. Nat Methods.

[40] Rezaei-Ghaleh N, Giller K, Becker S, Zweckstetter M (2011). Effect of zinc binding on β-amyloid structure and dynamics: implications for Aβ aggregation. Biophys J.

[41] Ashkenazy H, Abadi S, Martz E, Chay O, Mayrose I, Pupko T, Ben-Tal N (2016). ConSurf 2016: an improved methodology to estimate and visualize evolutionary conservation in macromolecules. Nucleic Acids Res.

[42] Drozdetskiy A, Cole C, Procter J, Barton GJ (2015). JPred4: a protein secondary structure prediction server. Nucleic Acids Res.

[43] Connor JH, Weiser DC, Li S, Hallenbeck JM, Shenolikar S (2001). Growth arrest and DNA damage-inducible protein GADD34 assembles a novel signaling complex containing protein phosphatase 1 and inhibitor 1. Mol Cell Biol.

[44] Rojas M, Vasconcelos G, Dever TE (2015). An eIF2α-binding motif in protein phosphatase 1 subunit GADD34 and its viral orthologs is required to promote dephosphorylation of eIF2α. Proc Natl Acad Sci USA.

[45] Brito Querido JB, Sokabe M, Kraatz S, Gordiyenko Y, Skehel JM, Fraser CS, Ramakrishnan V (2020). Structure of a human 48S translational initiation complex. Science.

[46] Beilsten-Edmands V, Gordiyenko Y, Kung JC, Mohammed S, Schmidt C, Robinson CV (2015). eIF2 interactions with initiator tRNA and eIF2B are regulated by post-translational modifications and conformational dynamics. Cell Discov.

[47] Ito T, Marintchev A, Wagner G (2004). Solution structure of human initiation factor eIF2alpha reveals homology to the elongation factor eEF1B. Structure.

[48] Abdulkarim B, Nicolino M, Igoillo-Esteve M, Daures M, Romero S, Philippi A, Seneé V, Lopes M, Cunha DA, Harding HP (2015). A missense mutation in PPP1R15B causes a syndrome including diabetes, short stature, and microcephaly. Diabetes.

[49] Kernohan KD, Tétreault M, Liwak-Muir U, Geraghty MT, Qin W, Venkateswaran S, Davila J, Holcik M, Majewski J, Care4Rare Canada Consortium (2015). Homozygous mutation in the eukaryotic translation initiation factor 2alpha phosphatase gene, PPP1R15B, is associated with severe microcephaly, short stature and intellectual disability. Hum Mol Genet.

[50] Holland PM, Cooper JA (1999). Protein modification: docking sites for kinases. Curr Biol.

[51] Mellor H, Proud CG (1991). A synthetic peptide substrate for initiation factor-2 kinases. Biochem Bioph Res Co.

[52] Taylor SS, Haste NM, Ghosh G (2005). PKR and eIF2α: integration of kinase dimerization, activation, and substrate docking. Cell.

[53] Luh LM, Bertolotti A (2020). Potential benefit of manipulating protein quality control systems in neurodegenerative diseases. Curr Opin Neurobiol.

[54] Costa-Mattioli M, Walter P (2020). The integrated stress response: from mechanism to disease. Science.

[55] Tsaytler P, Harding HP, Ron D, Bertolotti A (2011). Selective inhibition of a regulatory subunit of protein phosphatase 1 restores proteostasis. Science.

[56] Das I, Krzyzosiak A, Schneider K, Wrabetz L, D’Antonio M, Barry N, Sigurdardottir A, Bertolotti A (2015). Preventing proteostasis diseases by selective inhibition of a phosphatase regulatory subunit. Science.

[57] Krzyzosiak A, Sigurdardottir A, Luh L, Carrara M, Das I, Schneider K, Bertolotti A (2018). Target-based discovery of an inhibitor of the regulatory phosphatase PPP1R15B. Cell.

[58] Dalla Bella ED, Bersano E, Antonini G, Borghero G, Capasso M, Caponnetto C, Corbo M, Filosto M, Giannini F (2021). The unfolded protein response in amyotrophic later sclerosis: results of a phase 2 trial. Brain.

[59] Johnson D, Cohen P, Chen MX, Chen YH, Cohen PTW (1997). Identification of the regions on the M110 subunit of protein phosphatase 1M that interact with the M21 subunit and with myosin. Eur J Biochem.

[60] Jaravine VA, Zhuravleva AV, Permi P, Ibraghimov I, Orekhov VYu (2008). Hyperdimensional NMR spectroscopy with nonlinear sampling. J Am Chem Soc.

[61] Lee W, Tonelli M, Markley JL (2015). NMRFAM-SPARKY: enhanced software for biomolecular NMR spectroscopy. Bioinformatics.

[62] Jung YS, Zweckstetter M (2004). Mars – robust automatic backbone assignment of proteins. J BioMol Nmr J Biomol NMR.

[63] Altschul SF, Madden TL, Schäffer AA, Zhang J, Zhang Z, Miller W, Lipman DJ (1997). Gapped BLAST and PSI-BLAST: a new generation of protein database search programs. Nucleic Acids Res.

[64] Katoh K, Standley DM (2013). MAFFT multiple sequence alignment, software version 7: improvements in performance and usability. Mol Biol Evol.

[65] Clamp M, Cuff J, Searle SM, Barton GJ (2004). The Jalview Java alignment editor. Bioinformatics Oxf Engl.

[66] Mirdita M, Schütze K, Moriwaki Y, Heo L, Ovchinnikov S, Steinegger M (2022). ColabFold: Making protein folding accessible to all. Nature methods.

[67] Pettersen EF, Goddard TD, Huang CC, Meng EC, Couch GS, Croll TI, Morris JH, Ferrin TE (2021). UCSF ChimeraX: structure visualization for researchers, educators, and developers. Protein Sci.

[68] Perez-Riverol Y, Csordas A, Bai J, Bernal-Llinares M, Hewapathirana S, Kundu DJ, Inuganti A, Griss J, Mayer G, Eisenacher M (2019). The PRIDE database and related tools and resources in 2019: improving support for quantification data. Nucleic Acids Res.

[69] Gibson DG, Young L, Chuang RY, Venter JC, Hutchison CA, Smith HO (2009). Enzymatic assembly of DNA molecules up to several hundred kilobases. Nat Methods.

[70] Xiao JH, Davidson I, Matthes H, Garnier JM, Chambon P (1991). Cloning, expression, and transcriptional properties of the human enhancer factor TEF-1. Cell.

[71] Kenner LR, Anand AA, Nguyen HC, Myasnikov AG, Klose CJ, McGeever LA, Tsai JC, Miller-Vedam LE, Walter P, Frost A (2019). eIF2B-catalyzed nucleotide exchange and phosphoregulation by the integrated stress response. Science.

[72] Kashiwagi K, Yokoyama T, Nishimoto M, Takahashi M, Sakamoto A, Yonemochi M, Shirouzu M, Ito T (2019). Structural basis for eIF2B inhibition in integrated stress response. Science.

[73] Silva JC, Denny R, Dorschel CA, Gorenstein M, Kass IJ, Li GZ, McKenna T, Nold MJ, Richardson K, Young P (2005). Quantitative proteomic analysis by accurate mass retention time pairs. Anal Chem.

[74] Puchała W, Burdukiewicz M, Kistowski M, Dkşbrowska KA, Badaczewska-Dawid AE, Cysewski D, Dadlez M (2020). HaDeX: an R package and web-server for analysis of data from hydrogen-deuterium exchange mass spectrometry experiments. Bioinformatics.

[75] Delaglio F, Grzesiek S, Vuister GW, Zhu G, Pfeifer J, Bax A (1995). NMRPipe: A multidimensional spectral processing system based on UNIX pipes. J Biomol NMR.

[76] Lee W, Rahimi M, Lee Y, Chiu A (2021). POKY: a software suite for multidimensional NMR and 3D structure calculation of biomolecules. Bioinformatics.

[77] Kjaergaard M, Brander S, Poulsen FM (2011). Random coil chemical shift for intrinsically disordered proteins: effects of temperature and pH. J Biomol NMR.

[78] Kjaergaard M, Poulsen FM (2011). Sequence correction of random coil chemical shifts: correlation between neighbor correction factors and changes in the Ramachandran distribution. J Biomol NMR.

[79] Schwarzinger S, Kroon GJA, Foss TR, Chung J, Wright PE, Dyson HJ (2001). Sequence-dependent correction of random coil NMR chemical shifts. J Am Chem Soc.

[80] Tjandra N, Szabo A, Bax A (1996). Protein backbone dynamics and 15N chemical shift anisotropy from quantitative measurement of relaxation interference effects. J Am Chem Soc.

