# Supplementary material for: Recruitment of trimeric eIF2 by phosphatase non-catalytic subunit PPP1R15B
Source: Mol Cell. Author manuscript; Available in PMC 2024 Feb 28. (PMC7615683; doi:10.1016/j.molcel.2023.12.011)
Supplement: Supplementary material [file EMS194229-supplement-Supplementary_material.pdf]

Molecular Cell, Volume *84*

Supplemental information

Recruitment of trimeric eIF2 by phosphatase non-catalytic subunit PPP1R15B

Agnieszka Fatalska, George Hodgson, Stefan M.V. Freund, Sarah L. Maslen, Tomos Morgan, Sigurdur R. Thorkelsson, Marjon van Slegtenhorst, Sonja Lorenz, Antonina Andreeva, Laura Donker Kaat, and Anne Bertolotti

Molecular Cell, Volume *84*

Supplemental information

Recruitment of trimeric eIF2 by phosphatase non-catalytic subunit PPP1R15B

Agnieszka Fatalska, George Hodgson, Stefan M.V. Freund, Sarah L. Maslen, Tomos Morgan, Sigurdur R. Thorkelsson, Marjon van Slegtenhorst, Sonja Lorenz, Antonina Andreeva, Laura Donker Kaat, and Anne Bertolotti

Supplemental information

Figures S1-S6 and accompanying text

Table S2

Figure S1 Multiple sequence alignment of R15B orthologs. Related to Figure 1

Sequences were aligned using Mafft [76] and coloured according to Clustal [80] colouring scheme. Colour intensity indicates sequence conservation. A black arrow shows the beginning of the fragment used in this study.

Figure S2 HDX-MS analyses of eIF2 in the presence of R15B$^{414-613}$. Related to Figure 2

(A-C) Sequence coverage map in HDX-MS analyses of eIF2α (A), β (B) and γ (C) in presence of R15B$^{414-613}$. Bars represent the peptic peptides of eIF2 plotted against the amino acid sequence.

(D-L) Woods plots showing differences in deuterium uptake for eIF2 in the presence or absence of R15B$^{414-613}$. Deprotected, protected and non-significantly different peptides are in red, blue and grey respectively plotted as Δ fraction exchanged (Y-axis). Bar length corresponds to peptide length plotted against the sequence position (X-axis). Dashed and dotted lines indicate 98 and 99% confidence intervals applied to identify peptides with statistically significant deuteration differences. Data for eIF2α 3 sec, 30 sec, 50 min; eIF2β 30 sec, 5 min, 50 min and eIF2γ 30 sec, 5 min and 50 min are shown in panels (D-L) respectively. Error bars denote combined uncertainty of peptide deuteration calculated based on triplicate experiments.

Figure S3 HDX-MS analyses for R15B$^{414-613}$. Related to Figure 3

(A) Plot showing HDX for a given R15B$^{414-613}$ peptide following 3 sec incubation with D_2O. Bars indicate the peptic peptides of R15B$^{414-613}$ plotted against the amino acid sequence (X-axis). Y-axis: fraction of deuteration peptide compared to maximum level of measured deuteration. Error bars represent combined uncertainty calculated on triplicate experiments.

(B) Sequence coverage of R15B$^{414-613}$ in presence of eIF2 in HDX analyses. Bars indicate the peptic peptides of R15B$^{414-613}$ plotted on the amino acid sequence.

Figure S4 ^{15}N backbone relaxation of R15B$^{414\text{-}613}$ alone and in the presence of 10% eIF2. Related to Figure 4.

(A-E) Experimental ^{15}N backbone relaxation data for R15B$^{414\text{-}613}$ at 281.5K.

(G-K) Experimental ^{15}N backbone relaxation data for R15B$^{414\text{-}613}$ in the presence of eIF2 at 281.5K.

(A, G) $\{^1H\}$-^{15}N NOE,

(B, H) R_2

(C, I) η_{xy}

(D, J) R_1

(E, K) Superposition of measured R_2 (black line) and calculated exchange free R_2^0 (blue). Exchange contribution (Rex, red line).

(F) Peak intensity ratio of ^{1}H,^{15}N 2D spectra of R15B$^{414\text{-}613}$ on its own and after addition of 10% eIF2, only a modest degree of local attenuation was observed.

Figure S5 Sequence alignments of R15B$^{414\text{-}639}$. Related to Figure 5

(A) Pair-wise sequence alignments of the human R15B sequence repeats.

(B) Multiple sequence alignments of the first R15B repeat and the homologous sequence repeats identified in human R15A.

(C) Same as in (B) with the addition of R15 from Drosophila melanogaster (dm), ICP34 from the human Herpes simplex virus 1 (hhv1) and DP71L from the African swine fever virus (asfv).

Figure S6 pLDDT of R15B-eIF2 AlphaFold2 model. Related to Figure 7

(A) pLDDT scores shown for R15B$^{414\text{-}613}$, eIF2α 1-315, eIF2β 167-333, eIF2γ 1-472 as a contiguous representation.

(B) As in (A) with R15B$^{414\text{-}500}$.

Table S2 Oligonucleotides used in this study. Related to STAR methods.

Figure S1

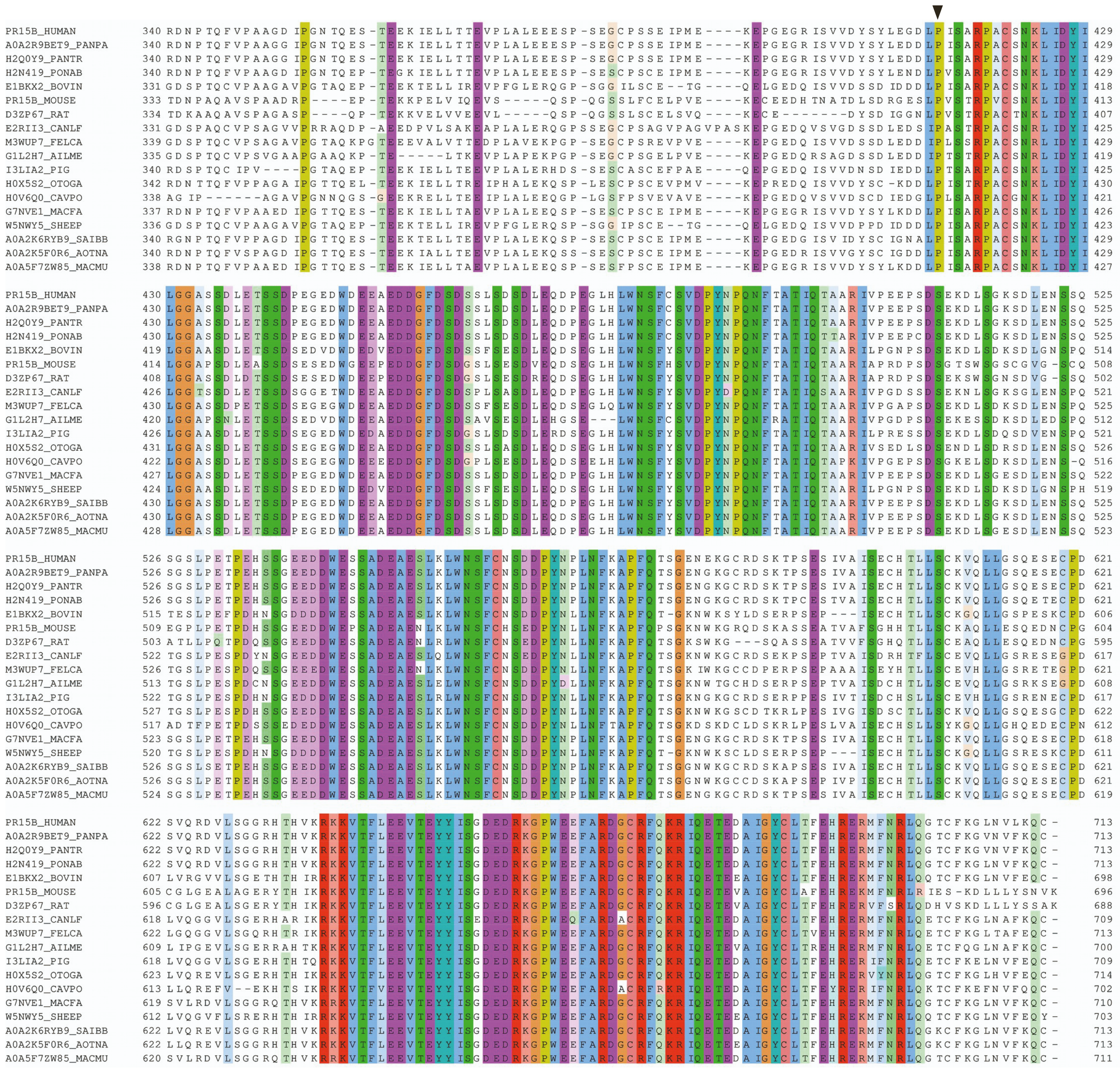
PR15B_HUMAN
A0A2R9BET9_PANPA
H2Q0Y9_PANTR
H2N419_PONAB
E1BKX2_BOVIN
PR15B_MOUSE
D3ZP67_RAT
E2RII3_CANLF
M3WUP7_FELCA
G1L2H7_AILME
I3LIA2_PIG
H0X5S2_OTOGA
H0V6Q0_CAVPO
G7NVE1_MACFA
W5NWY5_SHEEP
A0A2K6RYB9_SAIBB
A0A2K5F0R6_AOTNA
A0A5F7ZW85_MACMU

Figure S2

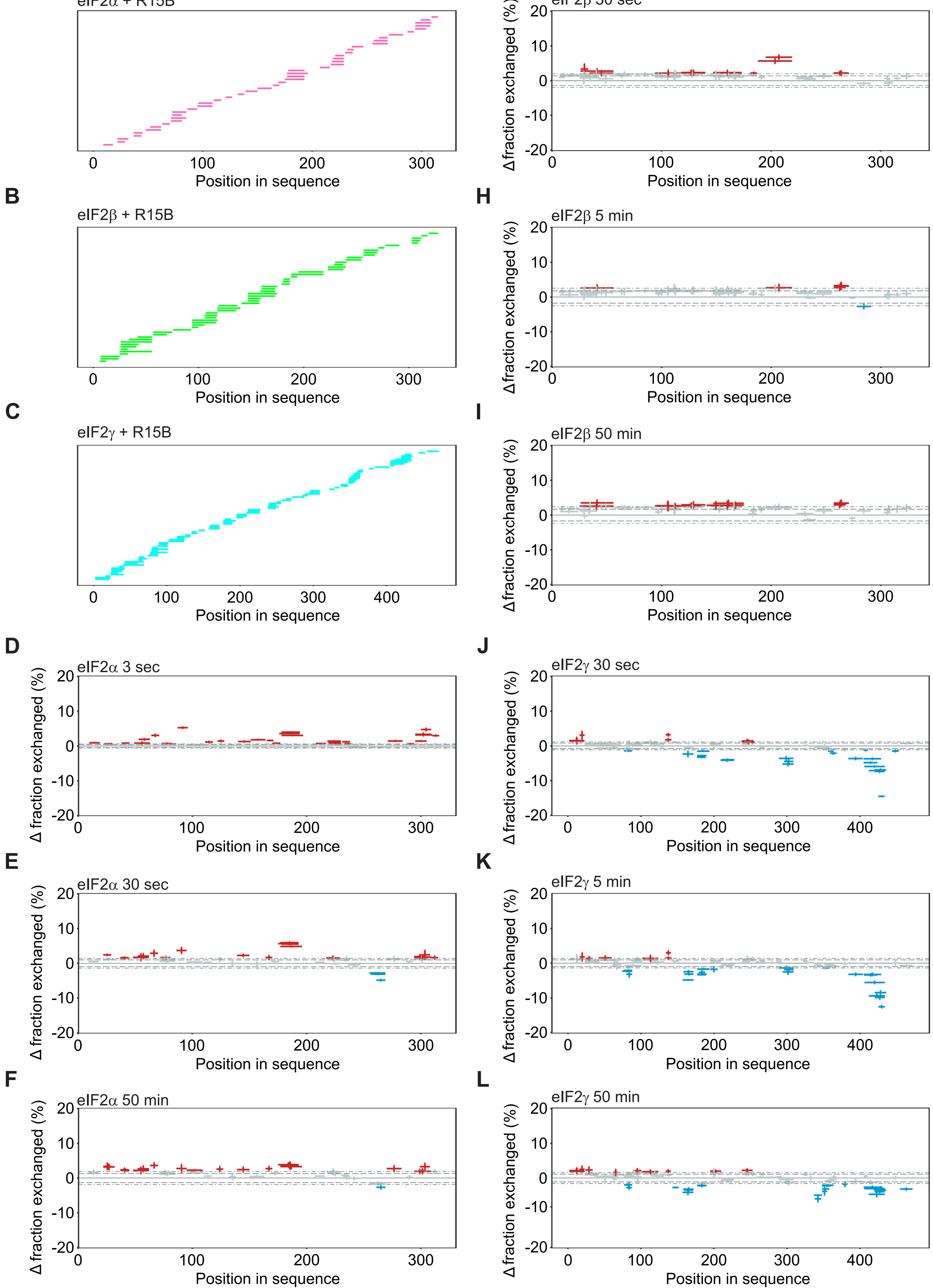
A
eIF2α + R15B
0
100
200
300
Position in sequence
B
eIF2β + R15B
0
100
200
300
Position in sequence
C
eIF2γ + R15B
0
100
200
300
400
Position in sequence
D
eIF2α 3 sec
E
eIF2α 30 sec
F
eIF2α 50 min
G
eIF2β 30 sec
H
eIF2β 5 min
I
eIF2β 50 min
J
eIF2γ 30 sec
K
eIF2γ 5 min
L
eIF2γ 50 min
Δ fraction exchanged (%)
20
10
0
-10
-20
Position in sequence

Figure S3

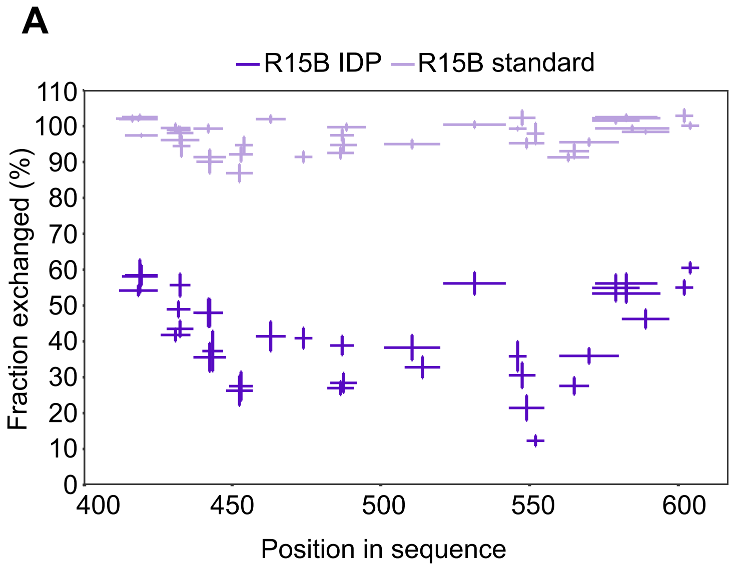
A
R15B IDP
R15B standard
Fraction exchanged (%)
110
100
90
80
70
60
50
40
30
20
10
0
400
450
500
550
600
Position in sequence

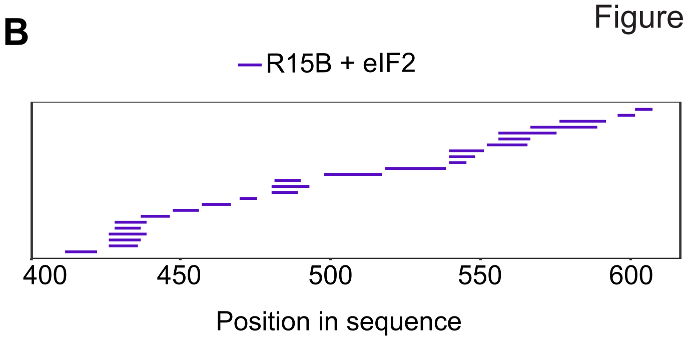
B
R15B + eIF2
400
450
500
550
600
Position in sequence

Figure S4

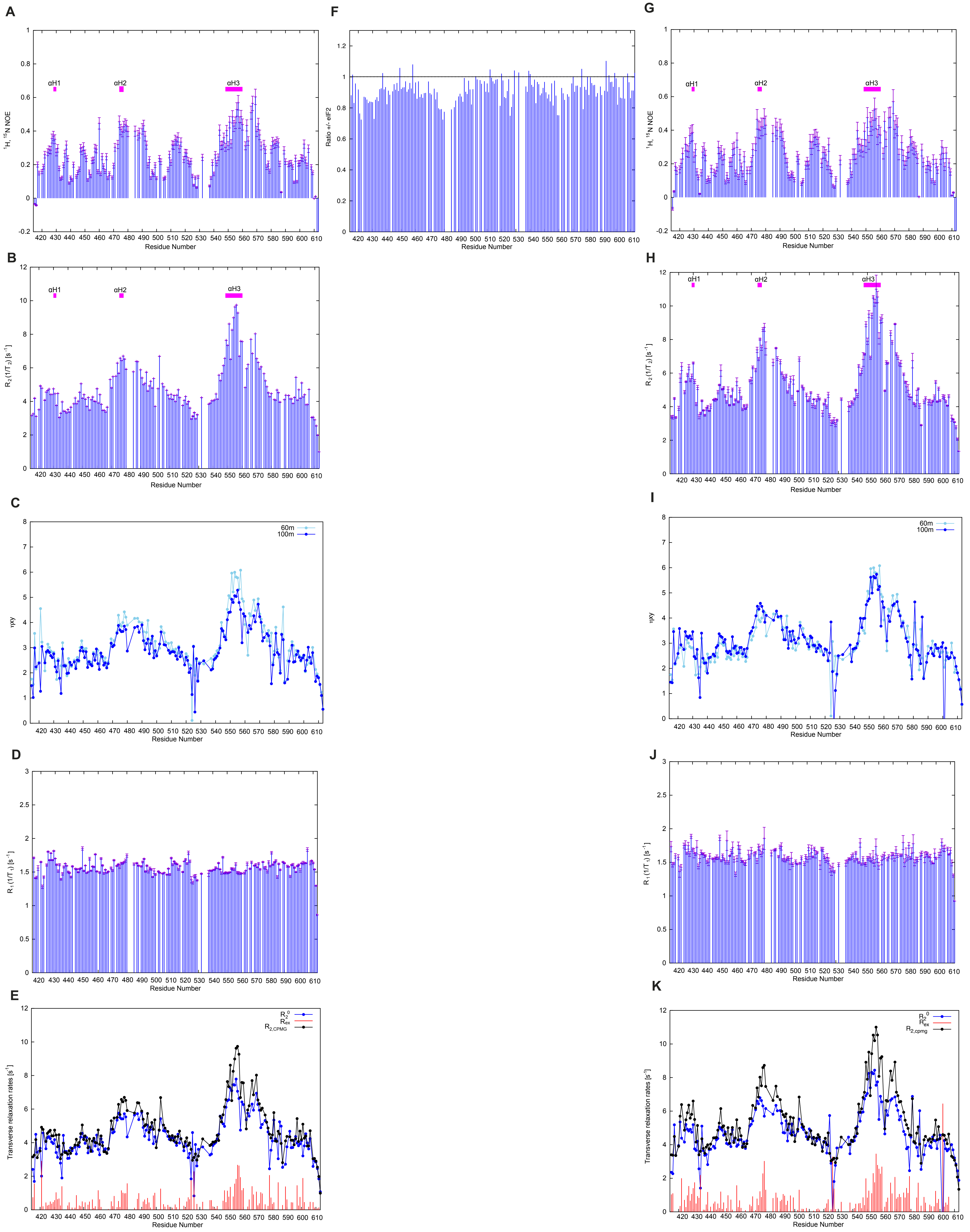

A
^{1}H, ^{15}N NOE
αH1
αH2
αH3
Residue Number
F
Ratio +/- eIF2
Residue Number
G
^{1}H, ^{15}N NOE
αH1
αH2
αH3
Residue Number
B
R_2 (1/T_2) [s^{-1}]
αH1
αH2
αH3
Residue Number
H
R_2 (1/T_2) [s^{-1}]
αH1
αH2
αH3
Residue Number
C
ηxy
60m
100m
Residue Number
I
ηxy
60m
100m
Residue Number
D
R_1 (1/T_1) [s^{-1}]
Residue Number
J
R_1 (1/T_1) [s^{-1}]
Residue Number
E
Transverse relaxation rates [s^{-1}]
R_2^0
R_{ex}
$R_{2,CPMG}$
Residue Number
K
Transverse relaxation rates [s^{-1}]
R_2^0
R_{ex}
$R_{2,cpmg}$
Residue Number

Figure S5

A

```
R15B   414  PISARPACSNKLIDYILGGASSDLETSSD----PEGEDWDEEAEDDGFDSDSSLSDSDLE  469
R15B   499  AARIVPEEPSDSEKDL--SGKSDLENSSQSGSLPETPEHSSGEEDD-WESSA--------  547

R15B   470  QDPEGLHLWNSFC-SVDPYNPQNFTATIQT  498
R15B   548  DEAESLKLWNSFCNSDDPYNPLNFKAPFQT  577
```

B

```
R15B   415  ISARPACSNKLIDYILGGASSDLETSSDPEGEDWDE---EAEDDGFDSDSSLSDSDLE  469
R15A   329  PPCIPPPSAFLKAWVY------------WPGEDTEEEEDEEEDEDSDSGSDEEEGEAE  374
R15A   376  SSSTPATGVFLKSWVY------------QPGEDTEE----EEDEDSDTGSAEDEREAE  417
R15A   419  SASTPPASAFLKAWVY------------RPGEDTEE----EEDEDVDSEDKEDDSEAA  460
R15A   469  HPSHPDQRAHFRGWGY------------RPGKETE----EEE-------AAEDWGEAE  503
```

C

```
hs-R15B      415  ISARPACSNKLIDYILGGASSDLETSSDPEGEDWDE---EAEDDGFDSDSSLSDSDLE  469
hs-R15A      419  SASTPPASAFLKAWVY------------RPGEDTEE----EEDEDVDSEDKEDDSEAA  460
hs-R15A      376  SSSTPATGVFLKSWVY------------QPGEDTEE----EEDEDSDTGSAEDEREAE  417
hs-R15A      329  PPCIPPPSAFLKAWVY------------WPGEDTEEEEDEEEDEDSDSGSDEEEGEAE  374
hs-R15A      469  HPSHPDQRAHFRGWGY------------RPGKETEE---EEAAEDWGEAE--------  503
dm_R15       129  -CVPSSCYFFIDLHPH-------------HGFK-------DTAEDCPTSQASSDRNNN  165
hhv1_ICP34   54   -GPPPSCSLLLRQWLH------------VPESASDD----DDDDDWPD----------  84
asfv_ DP71L  44   IGTNPT-----LAFILGG------------NEDL------SDDSDWDENFSLENTL--  76
```

Figure S6

A

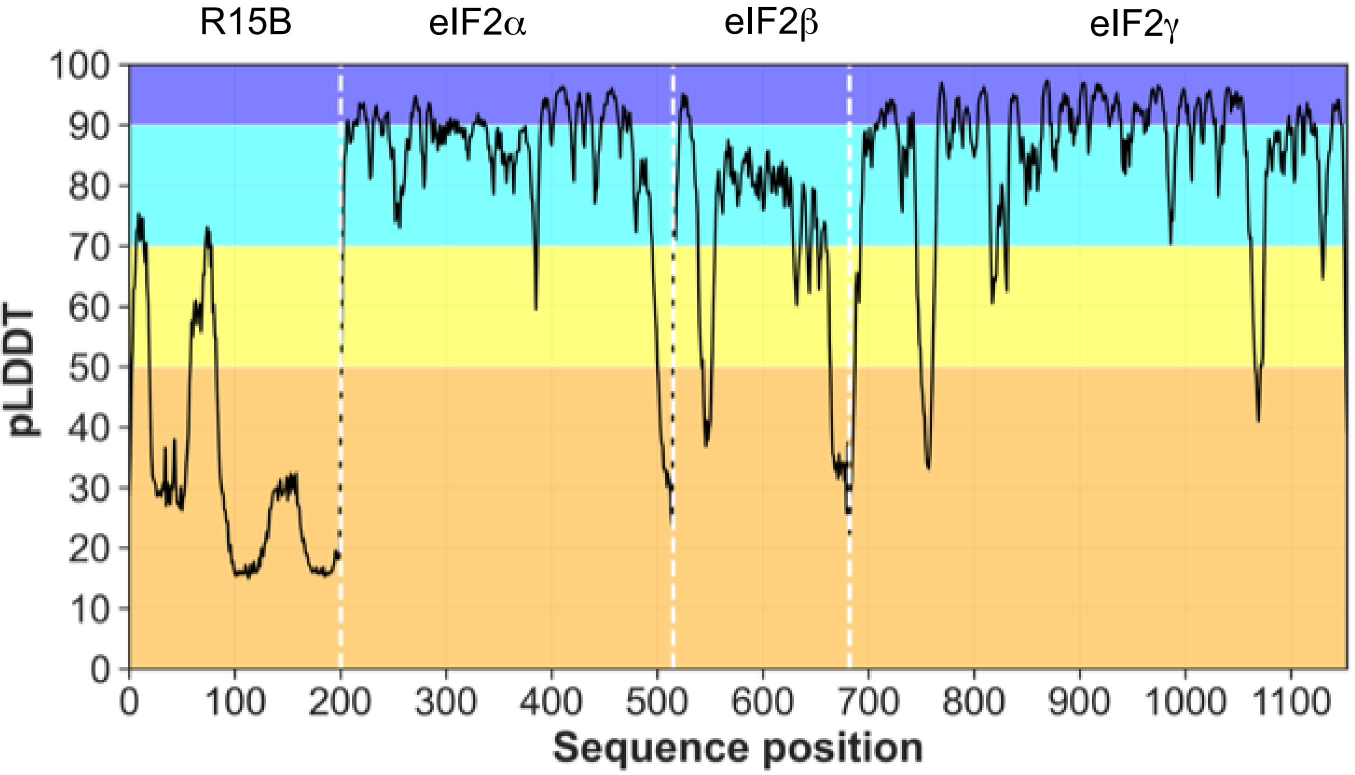
R15B
eIF2α
eIF2β
eIF2γ
pLDDT
0
10
20
30
40
50
60
70
80
90
100
0
100
200
300
400
500
600
700
800
900
1000
1100
Sequence position

B

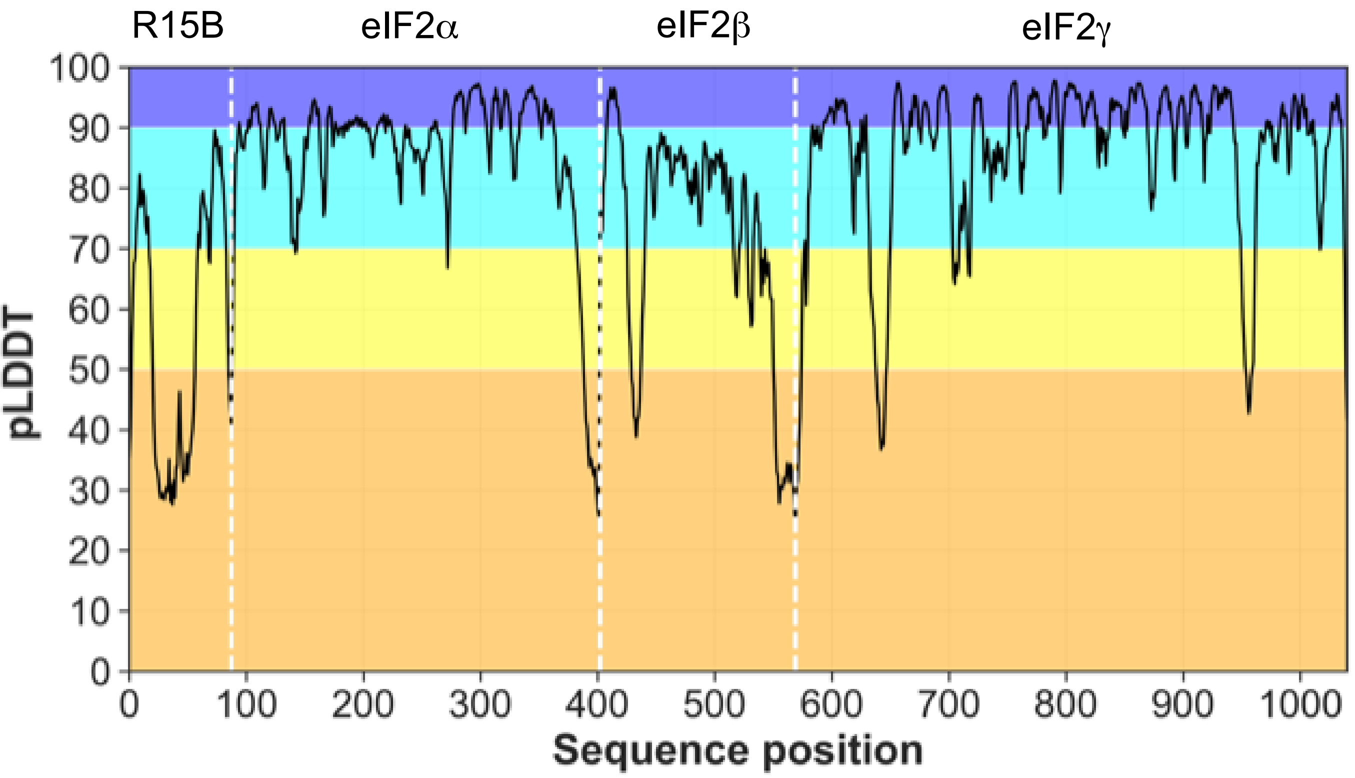
R15B
eIF2α
eIF2β
eIF2γ
pLDDT
0
10
20
30
40
50
60
70
80
90
100
0
100
200
300
400
500
600
700
800
900
1000
Sequence position

Table S2 : Oligonucleotides used in this study

Primer 5'-3'	SOURCE
R15B Forward aa1 PXJ41<br>ggatgacgacgataagATGGAGCCGGGGACAGGC	Sigma-Aldrich
R15B Forward aa411 PXJ41<br>ggatgacgacgataagGGTGACCTTCCCATTTCTGCCAGAC	Sigma-Aldrich
R15B Forward aa414 PXJ41<br>ggatgacgacgataagCCCATTTCTGCCAGACC	Sigma-Aldrich
R15B Reverse aa414 PXJ41<br>gtaccctcgagaactatcaGGGAAGGTCACCTTCTAGGTATGAGTAATCA	Sigma-Aldrich
R15B Reverse aa613 PXJ41<br>gtaccctcgagaactatcaCCCCAACAGCTGCACCTTACAAGAAAG	Sigma-Aldrich
R15B Reverse aa639 PXJ41<br>gtaccctcgagaactatcaTTTTCTTTTGACATGTGTGTG	Sigma-Aldrich
R15B N423D site directed mutagenesis Forward<br>AGACCAGCTTGTAGTGACAAACTGATAGATTAT	Sigma-Aldrich
R15B N423D site directed mutagenesis Reverse<br>ATAATCTATCAGTTTGTCACTACAAGCTGGTCT	Sigma-Aldrich
R15B Forward aa414 pET-47b<br>cagggacccggtCCCATTTCTGCCAGACCAGC	Sigma-Aldrich
R15B Forward aa632 pET-47b<br>cagggacccggtAGACACACACATGTCAAAAG	Sigma-Aldrich
R15B Reverse aa613 pET-47b<br>ggcaccagagcgttaCCCCAACAGCTGCACCTTAC	Sigma-Aldrich
R15B Reverse aa664 pET-47b<br>ggcaccagagcgttaCCATGGTCCTTTGCGATCCTC	Sigma-Aldrich
R15B Reverse aa705 pET-47b<br>ggcaccagagcgttaTTTGAAGCATGTTCCCTGGAGT	Sigma-Aldrich
